# Approaches for Long Lifetime Organic Light Emitting Diodes

**DOI:** 10.1002/advs.202002254

**Published:** 2020-11-12

**Authors:** Sujith Sudheendran Swayamprabha, Deepak Kumar Dubey, Shahnawaz, Rohit Ashok Kumar Yadav, Mangey Ram Nagar, Aayushi Sharma, Fu‐Ching Tung, Jwo‐Huei Jou

**Affiliations:** ^1^ Department of Materials Science and Engineering National Tsing Hua University Hsinchu 30013 Taiwan, Republic of China; ^2^ Birla Institute of Technology & Science‐Pilani Shamirpet‐Keesara Road, Jawahar Nagar, Shameerpet Hyderabad Telangana 500078 India; ^3^ Department of Solid State Lighting Technology Mechanical and Mechatronics Systems Research Labs. Industrial Technology and Research Institute Hsinchu 31057 Taiwan, Republic of China

**Keywords:** degradation, device architecture, lifetime, materials, OLED

## Abstract

Organic light emitting diodes (OLEDs) have been well known for their potential usage in the lighting and display industry. The device efficiency and lifetime have improved considerably in the last three decades. However, for commercial applications, operational lifetime still lies as one of the looming challenges. In this review paper, an in‐depth description of the various factors which affect OLED lifetime, and the related solutions is attempted to be consolidated. Notably, all the known intrinsic and extrinsic degradation phenomena and failure mechanisms, which include the presence of dark spot, high heat during device operation, substrate fracture, downgrading luminance, moisture attack, oxidation, corrosion, electron induced migrations, photochemical degradation, electrochemical degradation, electric breakdown, thermomechanical failures, thermal breakdown/degradation, and presence of impurities within the materials and evaporator chamber are reviewed. Light is also shed on the materials and device structures which are developed in order to obtain along with developed materials and device structures to obtain stable devices. It is believed that the theme of this report, summarizing the knowledge of mechanisms allied with OLED degradation, would be contributory in developing better‐quality OLED materials and, accordingly, longer lifespan devices.

## Graphical abstract

This work provides an in‐depth overview of the state of art of stability of organic light emitting diodes and covers important degradation issues involved in this technology. Several intrinsic and extrinsic degradation mechanisms within the materials and evaporator chambers are thoroughly analyzed and discussed.

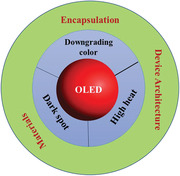

## 1 Introduction

### 1.1 World Revenue of Display and Lighting

Organic light emitting diodes (OLEDs) are at the cusp of becoming the dominant technology for high‐quality flat panel display as well as for solid‐state lighting owing to its unique disruptive features such as energy‐saving, wide view‐angle, fast response, high contrast, and high color purity. OLEDs are emitting flat, plan, diffused and soft light and producing eye‐catching images that have the class of natural light color, making them an ideal candidate for high‐quality flat panel display.^[^ ^1^, ^2^, ^3^, ^4^, ^5^, ^6^, ^7^, ^8^, ^9^, ^10^, ^11^, ^12^, ^13^, ^14^ ^]^ On the other hand, LEDs have to undergo multilevel “processing” in order to get close to the light quality offered by the OLED based counterparts. It is also difficult to obtain even dispersal into a near‐plane light source in LEDs.^[^ ^15^ ^]^ Similarly for general lighting purposes in offices or reading areas, which are usually larger than an average‐sized room, uniformly illuminated lighting sources are required. This requirement can be fulfilled by using almost similar size OLED lighting panels. However, this proportionately increases the manufacturing costs of the components which make this technology less feasible for general lighting purpose.

OLED technology also offers new approaches to be fabricated on flexible and stretchable substrates and its thinness, flexibility and extraordinary durability during worst mechanical conditions such as bending and twisting making it suitable for wearable electronics, biomedical appliances, electronic skins, and robotics. OLED components are capable of emitting stable and transformable light which can be tuned by the device architecture. The color and color temperature of OLEDs can be adjusted along with a wide range from 1500 to 20000 K with almost no restrictions, which generate the possibility of mimicking natural light style artificial light.^[^ ^16^ ^]^ Both OLED researchers and market analysts are strongly believed that the OLED lighting market will, sooner or later, take up a major portion of revenue and eventually disrupt the conventional technologies.

**Figure 1:**
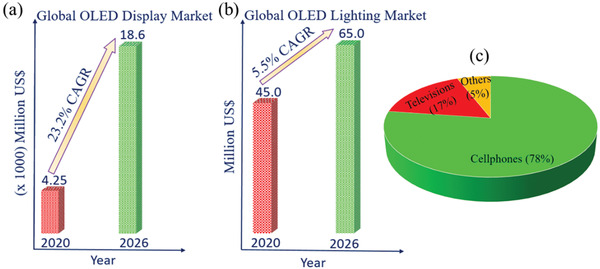
Overview of OLED global market revenue by a) display and b) lighting application. c) Contribution of OLED technology by application in next‐generation electronic devices.

According to Global OLED Display Market reported the global OLED Display market is valued at 42 490 million US$ in 2020 and is expected to reach 185 830 million US$ by the end of 2026, growing at a compound annual growth rate (CAGR) of 23.2% during 2021–2026 (**Figure** **1**a).^[^ ^17^ ^]^ Similarly, the global OLED Lighting Panels market is valued at 45 million US$ in 2020 and is expected to reach 65 million US$ by the end of 2026, growing at a CAGR of 5.5% during 2021–2026 (Figure 1b).^[^ ^18^ ^]^

The informative display is a key tool for information dissemination and human–machine communication and become an indispensable part of our lifestyle and gradually shaping it. The machine offers data to the user via the display, to which the user responds by providing feedback to the machine. The market of OLED displays has grown rapidly and has started to challenge other existing major technology owing to its potential applications in smartphones, tablets, computer monitors, televisions (TVs), automobile, head‐up‐display, smart watches, and so on. As per IDTechEx report, OLEDs for cellphones and TVs dominate the OLED sector, comprising 78% and 17% of the OLED market revenue in 2020, respectively (Figure 1c). Despite being 17% of the revenue, OLED TVs are 43% of the OLED market by area of the display. The third largest OLED application is wearables, which is 2% of the total OLED display market value and 0.3% by area in 2020.^[^ ^19^ ^]^ OLED display can be divided into two categories on the basis of driving mode, i.e., passive matrix OLED (PMOLED) and active matrix OLED (AMOLED). Among, AMOLED is superior because of its low weight, thickness, wide color gamut, and fast response time. It is also the most widely used large‐sized display in OLED sector with potential applications in cellphones, tablets and TVs.^[^ ^20^, ^21^ ^]^ However, it is criticized for a poor lifetime when compared with other counterparts, i.e., PMOLED.^[^ ^20^, ^21^ ^]^

Nowadays, the human being is spending their maximum time in an environment that is created by artificial lighting. It is used everywhere, including our home, school, office, shopping malls, factories as well as also for other outdoor purposes. According to the International Energy Agency's 2006 report, lighting consumes about 20% of total generated electric energy and 30–40% of total consumption of this energy in residential buildings and offices.^[^ ^22^ ^]^ Moreover, energy‐inefficient light sources such as incandescent bulbs are still lighting majors in many developing countries. In order to solve the energy crisis, both academics and industries have made a considerable effort to devise energy saving and long‐lasting lighting sources are in demand to solve the energy crisis. Amongst, all the existing lighting majors still LED is more dominating technology, but in the last few years OLED based lighting sources also show a great potential owing to its energy saving and other physiological features. Although a notable development has been made in OLED lighting, but still there are several challenges to realize such as high cost and performance at high brightness.^[^ ^23^, ^24^, ^25^, ^26^ ^]^ In lighting, several companies have launched trial products such as: Osram in Germany, Philips in the Netherlands, Visionox in the mainland, Lumiotec in Japan, and GE in the United States. Kaneka, LG, Samsung, Konica Minolta, etc., are also some noted companies.

**Figure 2:**
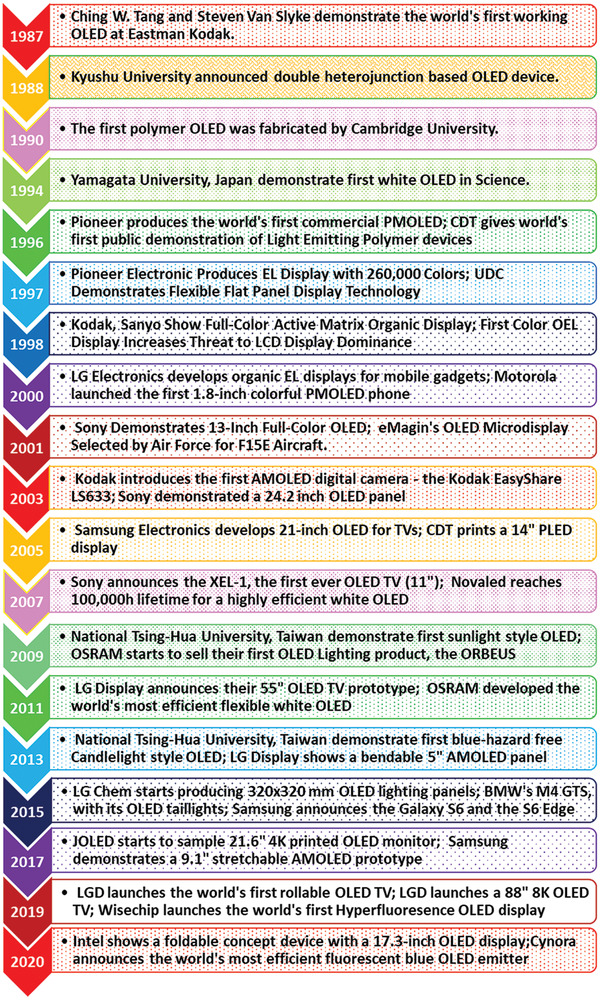
A brief history of OLED technology evolution.

OLED are used today to make efficient and beautiful lighting panels. Moreover, it is the only technology that can create large “area” flexible and transparent lighting panels. In 2016, Audi launched its first automobile with OLED lighting with the help of OSRAM. Furthermore, in 2018, Audi A8 uses four small vertical OLED taillight modules on each side, that are provided by Hella.^[^ ^27^ ^]^ In 2018, Acuity Brands launched 4′, 6′, and 8′ lamp based on OLED technology and named it the “Peerless OLE4 Olessence.”^[^ ^28^ ^]^ In 2019, they further launched the slimmer version of Olessence with the help of OLEDWorks.^[^ ^28^ ^]^ Despite being efficient and beautiful, OLED lighting also offers many physiological features. In this regard, in December 2018, First‐o‐lite, together with NTHU, launched the first candlelight OLED desk lamp that is blue hazard free as well as also free from Hg, glare and flicker, and providing a sensational pleasant environment.^[^ ^29^, ^30^, ^31^ ^]^ A complete chart of OLED development history has been demonstrated in the **Figure** **2**.

### 1.2 Challenges of OLED

OLEDs are conceptually different from most of the current lighting and display majors and offer arrange of attractive features such as high efficiency, low power consumption, fast switching, wide viewing angle, light weight, and flexibility.^[^ ^32^, ^33^ ^]^ Because of sustainable development in material chemistry, device physics and manufacturing technology, OLEDs are continuously improving in terms of performance, durability, and manufacturability, and many products based on it had already come into our daily life. But still this technology is suffering from some serious drawbacks, like the reduced life expectation given the extreme sensitiveness to oxygen and moisture as well as difficulties into encapsulate devices which cause an aggravation in the degradation that making the devices very sensitive and not efficient.^[^ ^33^ ^]^ This sensitiveness demands highly controlled production environment, which requires complex methods of fabrication and an increased production cost. Furthermore, roughness of the metal electrodes, poor interfacial bonding between organic and inorganic layer and the migration of the metal ions into the organic layers from the electrodes are also highly responsible for device efficiency and lifetime.

Besides the aforementioned issue, OLED's device physicists also have to answer some other questions related to the thermodynamics of OLED material like which parameters are responsible for their degradation, solubility, and proper dispersion in the phases that they're in. OLED's material chemists are also spending a lot of times on the molecular design of emitters.

### 1.3 Growth of OLED Paper and Patent

**Figure 3:**
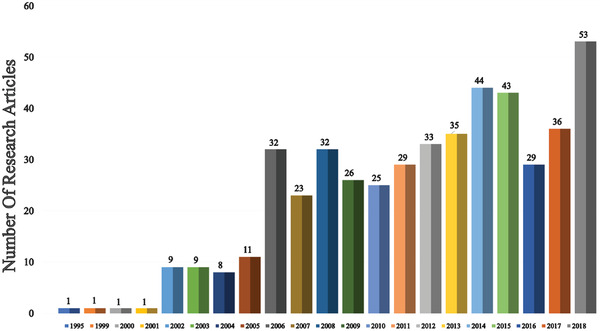
Number of research articles on WOLED per year (Web of Science).

**Figure 4:**
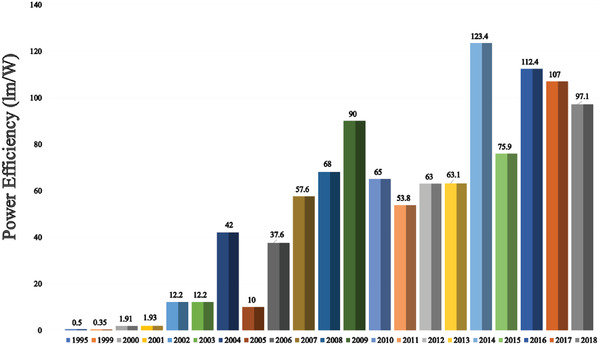
Highest power efficiency WOLED per year (Web of Science).

**Figure 5:**
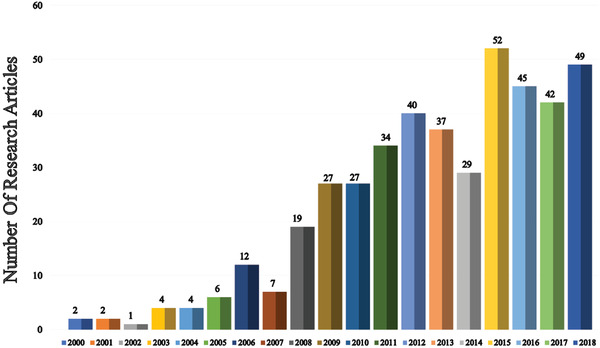
Number of research articles on blue OLED per year (Web of Science).

**Figure 6:**
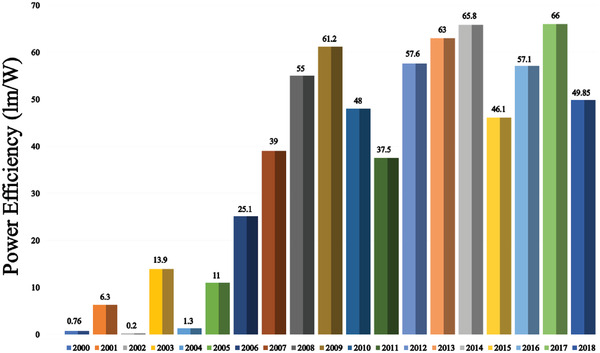
Highest power efficiency of blue OLED per year (Web of Science).

**Figure 7:**
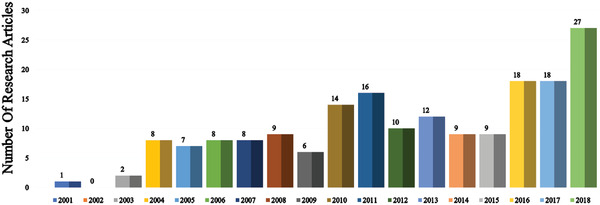
Number of research articles on red OLED per year (Web of Science).

**Figure 8:**
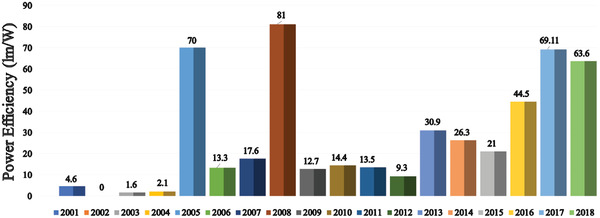
Highest power efficiency of red OLED per year (Web of Science).

**Figure 9:**
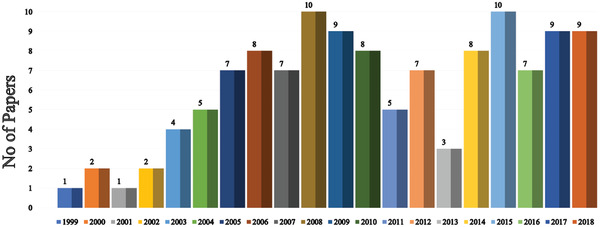
Number of papers of lifetime per year (Web of Science).

**Figure 10:**
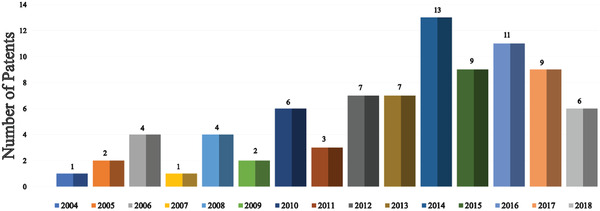
Illustrates the number of patents on WOLEDs filed per year (Web of Science).

Since the unveiling of the first OLED in 1987, numerous strategies have been applied to improve device efficiency and lifetime. In this section (**Figures** **3**–**10**), we are trying to provide clear statistics about the number of papers and patents growth curve per year for the particular color of OLEDs and the highest efficiencies reported per year for a specific color of OLED.

Figure 3 shows the number of publications on white OLEDs (WOLEDs) from 1995 to 2018. After the invention of first WOLED by Kido et al. in 1994, many research groups are focusing to improve the OLEDs efficiency and lifetime.^[^ ^34^ ^]^ Moreover, several studies dealing with the tuning of Commission Internationale de L'Eclairage (CIE) coordinates, color rendering index (CRI), spectrumresemblance index (SRI), and color temperature (CT) in WOLEDs.^[^ ^23^ ^]^ Till 2005, there is a moderate growth in WOLED development but later it gains huge attention from the academia and industry due to the successive advancement in RGB emitters. Hence, there is a tremendous increment in the number of WOLED publications after 2005. In 2018, 53 papers were published, describing the materials and methods for the production of white OLEDs. It is very crucial to reduce the fabrication and material cost for WOLEDs to become a disruptive technology in the lighting market.

Figure 4 shows the highest power efficiency (PE) of WOLEDs per year. The PE of OLEDs depends on a number of critical factors like material property, fabrication technique, and device architecture, etc. In 1994, Kido et al. demonstrated the first multilayer WOLED, which exhibited a PE of 0.83 lm W^−1^.^[^ ^34^ ^]^ They have used three constructive emissive layers namely blue, green, and red with different carrier transporting properties. Till 2008, the overall device power efficiencies were considerably lower than 60–70 lm W^−1^ but in the year 2009, Reineke et al. demonstrated a WOLED device achieving PE of 90 lm W^−1^ via implementing a periodic out‐coupling structure.^[^ ^35^ ^]^ Later, in 2014, Liu et al. fabricated a flexible WOLED which exhibited a maximum PE of 101.3 lm W^−1^.^[^ ^36^ ^]^ In the same year, Ou et al. demonstrated a WOLED with the highest PE of 123.4 lm W^−1^ by utilizing multilayered energy cascading structure.^[^ ^37^ ^]^ In the year 2016, Xu et. al. demonstrated WOLED with maximum PE of 112.4 lm W^−1^ using quasi‐random out‐coupling.^[^ ^38^ ^]^ In 2017, Tong and group members reported an efficient light extraction of OLEDs on a fully solution‐processed flexible substrate and reported a PE of 107 lm W^−1^.^[^ ^39^ ^]^ In the same year, Wu et al. displayed a WOLED possessing PE of 105 lm W^−1^ without light out‐coupling enhancement technique.^[^ ^40^ ^]^ Recently, Ying et al. revealed a high‐efficiency hybrid WOLED by introducing ultrathin nondoped phosphorescent emitters in a blue exciplex host exhibited PE of 97.1 lm W^−1^.^[^ ^41^ ^]^ Ying et al. reported an efficient WOLED through managing triplet excitons in the emission layer and achieved a 95.3 lm W^−1^ efficiency.^[^ ^42^ ^]^

Figure 5 demonstrated the number of papers published on blue OLEDs in each year, the average number of papers per year is significantly increasing after 2009. Lighting and display industries facing critical issues concerning in the short operational lifetime of blue OLEDs. Very recently, Lee et al. reported a review article regarding the current status, encounters, and future viewpoint of blue OLEDs. The short lifetime of blue OLEDs mainly attributed to wide bandgap and long triplet exciton lifetime.^[^ ^43^ ^]^ It is very crucial to develop deep blue emitters with high efficiency and lifetime, which should follow the National Television System Committee (NTSC) standard color coordinates of (0.14, 0.08).^[^ ^44^ ^]^ After 2014, there is drastic progress in the development of blue OLEDs, which may be influenced by the 2014 Nobel prize for bright blue light LED.^[^ ^45^ ^]^ The OLED industries are still trying to develop efficient and long lifetime blue OLEDs from phosphorescent and thermally activated delayed flourescence (TADF) emitters.

Figure 6 shows the increment in the highest PE of blue OLEDs per year. Until 2005, the reported blue OLEDs PE was less than 15 lm W^−1^. In 2006, Padmaperuma et al. demonstrated a blue OLED with a maximum PE of 25.1 lm W^−1^ by utilizing a novel charge‐transporting host material.^[^ ^46^ ^]^ In 2008, Su's research group demonstrated blue electrophosphorescent OLED with a PE of 55 lm W^−1^ at 100 cd cm^−2^.^[^ ^47^ ^]^ In 2009, Bhansali et al. revealed a high efficient blue electrophosphorescence OLED exhibited PE of 61.2 lm W^−1^ via utilizing Pt(II)‐pyridyltriazolate complex as an emitter molecule.^[^ ^48^ ^]^ Through employing a pyridine containing electron transport layer, Ye et al. demonstrated blue OLED with maximum a PE of 65.8 lm W^−1^.^[^ ^49^ ^]^ In the year 2017, Sasabe et al. reported a blue OLED with a power efficiency of 66 lm W^−1^ through an isonicotinonitrile‐based novel TADF emitter.^[^ ^50^ ^]^

Figure 7 shows the number of papers based on red OLEDs per year. Red emission is one of the key components for displays, WOLEDs, and low CT OLEDs.^[^ ^31^, ^51^, ^52^ ^]^ Numerous studies were going on to improve the efficiency and lifetime of deep red, red, and orange‐red OLEDs. In 2018, 27 papers were discussed about red OLEDs. Red OLEDs are showing higher lifetime and efficiency compared to blue OLEDs. Fluorescent, phosphorescent, and TADF materials are utilizing to fabricate high efficiency and long lifetime red OLEDs. The color purity, efficiency, and long lifetime of red OLEDs strong influencing the durability of OLED displays.^[^ ^51^ ^]^

Figure 8 shows the highest PE of red OLED per year. In 2005, Wellmann et al. reported a highly efficient PIN red OLED, which exhibited a PE of 10.0 lm W^−1^ at 100 cd m^−12^ with CIE coordinates of (0.69, 0.31).^[^ ^53^ ^]^ In 2008, Meerheim et al. demonstrated a red OLED with a PE of 81 lm W^−1^ with an out‐coupling device mechanism through a microcavity amplification between the cathode and Ag layer.^[^ ^54^ ^]^ In 2017, Cui et al. displayed a red OLED with maximum PE of 69.11 lm W^−1^ based on terbium and gadolinium complexes as sensitizers.^[^ ^55^ ^]^ In 2018, Wang et al. demonstrated a high efficiency red phosphorescent OLED (PhOLED) with a PE of 63.6 lm W^−1^ by employing a novel TADF host material.^[^ ^56^ ^]^ In the same year, Wang et al. reported a double emissive layer based high efficiency OLED device with a PE 53.5 lm W^−1^.^[^ ^57^ ^]^

Li et al. reported organic shell‐molecule to avoid the transition problem of triplet in OLEDs.^[^ ^58^ ^]^ They fabricated OLEDs using stable neutral *π* radicals and achieved an EQE of 2.4%.^[^ ^58^ ^]^ They reported a new stable room‐temperature luminescent radical, (*N*‐carbazolyl)bis(2,4,6‐tirchlorophenyl)‐methyl radical (CzBTM), which exhibited deep red to near infrared emission.^[^ ^59^ ^]^ In another report, they demonstrated open‐shell, doublet red dopants that emit light after donor–radical charge transfer.^[^ ^60^ ^]^ In 2019, they reported a deep‐red/near‐infrared OLED with a maximum quantum efficiency of 5.3% with donor–acceptor neutral radicals not following the Aufbau principle.^[^ ^61^ ^]^ Tris(2,4,6‐trichlorophenyl)methyl–pyridoindolyl derivatives showed a high photoluminescence quantum yield of >90% and the device showed pure red emission with an EQE of 12%.^[^ ^62^ ^]^

Figure 9 illustrates the number of published papers focused on the OLEDs lifetime perspective. However, the OLEDs lifetime still remains the ongoing challenge in front of the researcher communities that repress it from present lighting and display technologies. In 2015, Scholz et al. published an informative review regarding the external and internal factors influencing the device lifetime.^[^ ^33^ ^]^ It's always crucial to solve these challenges associated with the device lifetime. Mainly, blue OLEDs facing unacceptably short lifetimes issues compared with red and green OLEDs.^[^ ^43^ ^]^

Every year a huge number of patents were filling based on the novel material, methods or technology perspective for improving the performance and lifetime of WOLEDs. Figure 10 illustrates the number of WOLEDs filed per year. Vast varieties of approaches were adopted to improve the efficiency of OLEDs compared to lifetime. Most of the publications and patents in OLED field are discussing the efficiency improvement. Despite the great achievements with device efficiency, an effective way to improve device lifetime is needed for the wider acceptance of OLED in display and lighting market. We need to focus on the development of devices with high efficiency as well as a long lifetime.

### 1.4 Ongoing Challenges

#### 1.4.1 Longer Lifetime at Higher Brightness

It is always crucial to obtain extended operational lifetime at higher brightness. The brightness of OLED devices increases with the applied current. With the increase in applied current, the lifetime of device decreasing. That means the lifetime of OLEDs is inversely proportional to the brightness, which is calculated as

(1) $$\begin{array}{c} L_{0}^{n}\times t_{1/2}=\mathrm{constant} \end{array}$$

where the *L*_0_ is the initial brightness, *t*_1/2_ is the time required to decay 50% of initial brightness (LT_50_), and *n* is the acceleration factor.

Achieving high brightness at low voltage can prevent the device degradation associated with higher current. Lee et al. reported an OLED exhibits high brightness at low voltage, 10 000 cd m^−2^ at 4 V. They achieved the same by applying high mobility electron transporting layer (ETL) material and mixed hosts.^[^ ^63^ ^]^ Recently, Kido's group reported a sterically bulky hole transporting material to attain a higher lifetime at high luminance. At 1000 cd m^−2^, the TADF device showed and EQE > 20%, 50 lm W^−1^ PE and LT_50_ of 10 000 h .^[^ ^64^ ^]^ It is necessary to develop OLEDs with a longer lifetime at higher brightness and lower voltage to save energy and money. **Table** **1** is showing the Universal Display Corporation (UDC) OLED material performance, which is adopted from Prof. S. R. Forrest presentation at American Physical Society (APS) March meeting 2018.^[^ ^65^ ^]^

**Table 1:** Universal Display Corporation (UDC) OLED material performance^[^ ^65^ ^]^

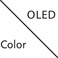	PhOLEDs		Fluorescent OLED	
	1931 CIE coordinates	*T*_50_ [h]	1931 CIE coordinates	*T*_50_ [h]
Red	(0.64, 0.36)	900 000	(0.67, 0.33)	160 000
Green	(0.31, 0.63)	400 000	(0.31, 0.63)	200 000
Blue	–	<100	(0.14, 0.12)	11 000

#### 1.4.2 Longer Lifetime at Elevated Temperatures

Both external and internal temperature influence the OLED lifetime and performance. Displays and mobile phone screens are exposed to different temperature ranges. Therefore, temperature management is crucial for the performance of OLEDs at high brightness. The device operating temperature influences both lifetime and efficiency.^[^ ^66^ ^]^ High heat generation may lead to the device meltdown or fracture. Thermal degradation is one of the major barriers to the production of longer lifetime OLED. Nowadays many techniques are employing to dissipate the heat energy associated with OLEDs.^[^ ^67^, ^68^, ^69^, ^70^ ^]^ We discussed the high heat caused device meltdown or substrate fracture and Joule heat in Sections 2.1.2 and 2.2.9.

#### 1.4.3 Longer Lifetime at Harsh Environments

Harsh environments associated with device fabrication and operating strong affecting the long lifetime operation. In 2016, Kaur et al. published a review paper discussing the influence of environmental factors on organic light emitting diode (OLED) displays.^[^ ^71^ ^]^ Presence of moisture, oxygen, and impurities strongly affecting the OLED lifetime, we are discussing the same issues in the Sections 2.2.1, 2.2.2, and 2.2.10. In 2016, Fujimoto et al. reported the influence of vacuum chamber impurities on the lifetime of OLEDs.^[^ ^72^ ^]^ The vacuum chamber impurities significantly influenced the lifetime of the experiment and reproducibility of the results. In order to achieve higher efficiency and longer lifetime, need to eliminate moisture, oxygen, and impurities mainly from the fabrication environment.

## 2 Degradation Phenomena and Failure Mechanisms

### 2.1 Degradation Phenomena

#### 2.1.1 Dark Spot

“Dark spots” or “black spots” are the nonemissive regions, which formed in the active area of OLEDs under the operating or storage conditions according to the studies by Scholz et al.,^[^ ^33^ ^]^ Turak et al.,^[^ ^73^ ^]^ Ke et al.,^[^ ^74^ ^]^ and Zardareh et al.^[^ ^75^ ^]^. Liew et al. and Fujihira et al. found the dark spots to form at the interfaces between the organic and conducting layers.^[^ ^76^, ^77^ ^]^ Güney et al. reported the growth of such dark spots under electrical stress.^[^ ^78^ ^]^ According to McElvain et al. and Aziz et al. efficiency, brightness, and lifetime of OLED decrease with the increase of dark spots.^[^ ^79^, ^80^ ^]^

The main external factor influencing the dark spot formation is the presence of humidity, dust particles, pinholes, spikes on ITO, short circuit, etc. In 2018, Azrain et al. reported a review article, which reviewed the mechanisms responsible for the formation of dark spots in OLEDs.^[^ ^81^ ^]^ The report summarized that the main reasons for dark spot formation are pinholes and high electrical current density. Weijer et al. reported that the penetration of water through pinholes in the cathode causes local oxidation of the cathode and which leads to the formation of nonemissive area in the device.^[^ ^82^ ^]^ Ohzu et al. calculated the dark spot formation in flexible OLEDs with the amount of water penetrated into the device.^[^ ^83^ ^]^ Ding et al. described the nature of catastrophic OLED lighting panel failure and the dark spot formation.^[^ ^84^ ^]^

**Figure 11:**
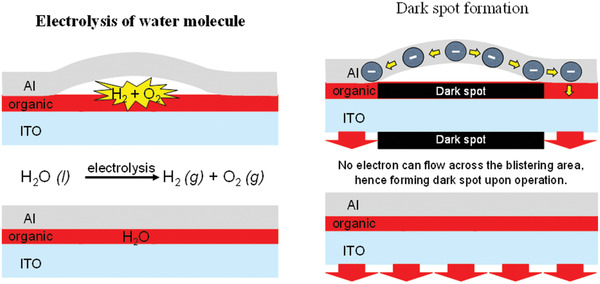
Electrolysis of water molecules and formation of dark spots inside an OLED device.

In the 1990s, the process of encapsulation was proposed as a solution for dark spot formation, thereby increasing the lifetime of the device. The different encapsulation techniques are discussed in Section 2.2.2. According to Ke et al. the roughening of the polymer/electrode interface because of metal migration increases the local current thereby leading to dark spot formation. It was further suggested to smoothen the polymer/electrode interface in order to prevent the dark spot formation.^[^ ^74^ ^]^ Chan et al. and Phatak et al. reported that elevated temperature deposition of cathode layer reduced the black dot formation because of the increase in the cathode/organic layer adhesion.^[^ ^85^, ^86^ ^]^ Liu et al. observed that the base etching process, without changing the ITO thickness and sheet resistance smoothens the anode (ITO) surface, which hence enhances the OLED lifetime by preventing dark spot formation.^[^ ^76^ ^]^ Liew et al. removed the cathode by scotch tape and the new cathode deposited immediately in a vacuum chamber, in order to prevent cathode delamination and dark spots.^[^ ^87^ ^]^ The invention of encapsulation technique was an important milestone in OLED technology, which effectively protects the device from oxygen and moisture. Minimization of dark spots will improve the device performance with effective brightness, efficiency and lifetime. The mechanism of dark spot formation shows in **Figure** **11**.

#### 2.1.2 High Heat Caused Device Melt Down or Substrate Fracture

Device meltdown or substrate fracture occurring mainly due to the high heat created inside the device. According to Chen et al. one of the major challenges facing by flexible OLEDs is fracture of particularly thin brittle conducting transparent oxide films, it may depend on the deposition temperature also.^[^ ^88^ ^]^ Production of Joule heat originates thermal expansion, interlayer diffusion of materials and crystallization or melting of organic materials, which reduce the lifetime of OLEDs. Regarding generation of Joule heat, after effects and reduction methods of Joule heating described in Section 2.2.8. Yan et al. introduced a polycarbonate high‐temperature capable substrate for organic electronics.^[^ ^89^ ^]^ Many studies are going on to defend the high heat caused problems in OLEDs.

#### 2.1.3 Downgrading Luminance and/or Color

Generally, the lifetime of an OLED is defined as the time required for the luminance to become 50% of the initial luminance.^[^ ^12^ ^]^ According to Van Slyke et al., luminance decay is coulombic, which is directly proportional to the current density applied.^[^ ^90^ ^]^ The luminance efficiency may be reduced by elevated temperature, accumulation of OLED degradation products, accumulation of immobile charge carriers, etc. Parker et al. reported that the luminance falloff increases with increase in temperature.^[^ ^91^ ^]^ According to him, higher temperature will change the morphology of polymeric materials and reducing the luminance efficacy. So et al. reported that accumulation of OLED degradation products affects luminance efficiency and operating voltage.^[^ ^92^ ^]^ He observed that formation of traps producing nonradiative recombination centers and reduce the luminance efficiency. Kondakov et al. reported, the accumulation of immobile charge carriers also affects the luminance efficiency.^[^ ^93^ ^]^Wang et al. conducted the constant‐brightness driving mode experiment to found the decay behavior, he divided the decay stages as i) linear increase, ii) exponential increase, and iii) vertical increase of current density over time.^[^ ^94^ ^]^

According to Aziz et al. luminance degradation occurs mainly through three degradation modes i) dark‐spot degradation, ii) catastrophic failure, and iii) intrinsic degradation.^[^ ^95^ ^]^ Ishii et al. described that the luminance decay happening via two steps; Initially the exponential luminance decay occurring due to chemical degradation and in the next step, rapid decline because of internal electric field.^[^ ^96^ ^]^ Young et al. reported that NPB^+^ (Radical ion of hole transporting material *N*,*N*′‐Bis(naphthalen‐1‐yl)‐*N*,*N*′‐bis(phenyl)benzidine, NPB) is an active quencher of blue luminescence via Förster energy transfer.^[^ ^97^ ^]^ OLEDs with tandem device structure are showing better efficiency and brightness.^[^ ^98^ ^]^ Lee et al. increased the luminance efficiency by introducing hole‐transporting interlayers between HTL and emissive layer (EML).^[^ ^99^ ^]^ Kitamura et al. improved the luminance efficiency by introducing SiO_2_/SiNx photonic crystals on ITO substrate.^[^ ^100^ ^]^ Li et al. introduced CNT templates as external electron source, which compensate the electron deficiency in the device and enhanced the luminance efficiency.^[^ ^101^ ^]^ Microlens array is an excellent method to improve luminance efficiency of OLED device.^[^ ^102^ ^]^

### 2.2 Failure Mechanisms and Plausible Solutions

#### 2.2.1 Moisture Attack

The presence of moisture in OLEDs will drastically reduce the device lifetime mainly via the formation and/or growth of dark spot formation, the decrease of electroluminescence (EL) intensity, the change in the electronic structure of organic layers, and the corrosion of cathode.^[^ ^103^, ^104^ ^]^ The penetration of moisture through materials can be conducted by the damp heat (DH) test as revealed in the “61215 Test” defined by the International Electrotechnical Commission (IEC).^[^ ^105^ ^]^ Peike et al. described that grid corrosion or reduced conductivity between the emitter and grid is the most likely cause of DH‐induced degradation.^[^ ^106^ ^]^ Laronde et al. employed the same DH testing to investigate the degradation of photovoltaic modules subjected to corrosion.^[^ ^107^ ^]^ According to Burrows et al., water and oxygen are the main factors influencing premature device failure.^[^ ^108^ ^]^ Liao et al. examined the effect of moisture on the electroluminescence intensity of OLEDs.^[^ ^103^ ^]^ He found that both the HTL and ETL were negatively affected by moisture, leading to dark spot formation and operational instability and hence device degradation. The same group reported the tris‐(8‐hydroxyquinoline) aluminum (Alq_3_) layer to undergo electronic structure change under the exposure of moisture.^[^ ^109^ ^]^

**Figure 12:**
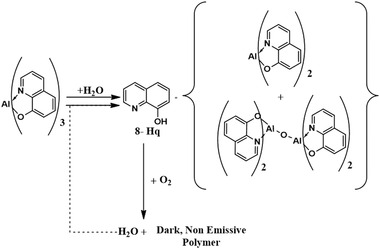
The reaction of Alq_3_with moisture and oxygen^[^ ^110^ ^]^

**Figure 13:**
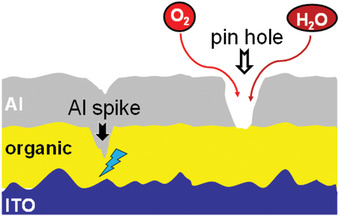
Pinholes in metal layer causing device failure.

Papadimitrakopoulos et al. explained the chemistry behind Alq_3_ degradation (**Figure** **12**) in the presence of moisture and oxygen.^[^ ^110^ ^]^ Aziz et al. reported the electrolysis reaction (Equation (1)) to occur in the Mg/Ag cathode due to the moisture adsorbed on the electrode surface.^[^ ^111^ ^]^ Lim et al. examined the effect of moisture on the dark spot formation, and they reported that the presence of pinholes enhanced the moisture attack by providing the undesirable penetration pathway.^[^ ^104^ ^]^ The moisture and oxygen penetration through pinholes is demonstrated in **Figure** **13**. Nevertheless, the most dependable effective approaches to reduce moisture attack is the employment of encapsulation, introduction of moisture seal, usage of desiccants, etc. Different types of encapsulation techniques are discussed in Section 2.2.2. Chun et al. reported a moisture seal containing alternating organic and inorganic layers, such as of epoxy and SiNH, to prevent moisture attack.^[^ ^112^ ^]^ Torres et al. studied different type desiccants for avoiding moisture attack.^[^ ^113^ ^]^ As observed, effective prevention of infiltration of moisture into the OLED device will increase both the device performance and lifetime

(2) $$\begin{array}{c} \mathrm{Cathode}\left(\mathrm{reduction}\right):\;2H_{2}O\left(l\right)+2e^{-}\to H_{2}\left(g\right)+2OH^{-}\left(\mathrm{ag}\right) \end{array}$$

(3) $$\begin{array}{c} \mathrm{Anode}\left(\mathrm{oxidation}\right):\;4OH^{-}\left(\mathrm{aq}\right)\to O_{2}\left(g\right)+2H_{2}O\left(l\right)+4e^{-} \end{array}$$

(4) $$\begin{array}{c} \mathrm{Total}\;\mathrm{reaction}:\;2H_{2}O\left(l\right)\to 2H_{2}\left(g\right)+O_{2}\left(g\right) \end{array}$$

#### 2.2.2 Oxidation

From the earlier days of organic electroluminescent device invention, researchers were trying to resolve the problems like oxidation and corrosion.^[^ ^114^ ^]^ Oxidation in OLED devices occurs mainly due to electrochemical reactions, moisture attack, and creates dark, nonemissive spots in device and limits the device lifetime.^[^ ^79^ ^]^ According to Juharia et al., carrier mobility of OLED materials could be destroyed by oxidation.^[^ ^115^ ^]^ According to Burrows et al., variations in the degree of wetting of organic surface by electrodes and defects formed during fabrication promotes oxidation.^[^ ^116^ ^]^ Do et al. reported that chemical oxidation of organic layers leads to evolution of heat and gases, which enhances device degradation by detachment of the electrode thin layer from organic/polymer layer.^[^ ^117^ ^]^ According to Schaer et al. the thermal diffusion of oxygen leads to the oxidation of both electrodes and organic layers.^[^ ^118^ ^]^ In order to prevent oxidation, Shen et al. introduced a metallic capping layer after the electrode.^[^ ^119^ ^]^ Nevertheless, avoidance of moisture will reduce the oxidation process in OLEDs. The encapsulation of OLED devices is a proficient method to prevent oxidation .

Various encapsulation techniques have been developed to reduce the influence of impurities on the overall performance of OLEDs. Some important encapsulation techniques adopted in OLED industry include Al_2_O_3_/ZrO_2_ nanolamination by Meyer et al.^[^ ^120^ ^]^ and Seo et al.,^[^ ^121^ ^]^ silica nanoparticle incorporated organic/inorganic nanocomposites by Jin et al.,^[^ ^122^ ^]^ graphene oxide nanocomposites by Jeon et al.,^[^ ^123^ ^]^ atomic layer deposition (ALD) of AlOx films by Park et al.,^[^ ^124^ ^]^ chemical vapor deposition of polymer films by Yamashita et al.,^[^ ^125^ ^]^ and siloxane or siloxane derivatives by Biebuyck et a1.,^[^ ^126^ ^]^ etc.

#### 2.2.3 Corrosion

The chemical corrosion is mainly happening due to the exposure of materials/electrodes to moisture, the mechanism described in Section 2.2.1. The corrosion processes in OLED affect electrodes^[^ ^127^ ^]^ or organic/polymer layers,^[^ ^128^ ^]^ which affects the OLED lifetime. According to Aziz et al. galvanic corrosion leads to bubble formation in OLED devices.^[^ ^128^ ^]^ Lin et al. reported that under electrical stress conditions, the presence of bases enhances both corrosion of polymers and bubble formation.^[^ ^129^ ^]^ According to Sierros et al., in flexible optoelectronics the ITO undergoes stress corrosion cracking, the rate of which would be higher in the presence of acids.^[^ ^130^ ^]^ Effective encapsulation methods can prevent the corrosion rate. According to Paetzold et al. in order to slow down the corrosion rate of different layers in OLED, needs to apply substrates with low permeation rate for air and water.^[^ ^131^ ^]^ Calcium corrosion test is extensively used for the determination of moisture permeation extent.^[^ ^132^ ^]^ Arai et al. introduced a gold doped Mg cathode, which prevents the nonemissive area formation due to corrosion.^[^ ^133^ ^]^

#### 2.2.4 Electron Induced Migrations

**Figure 14:**
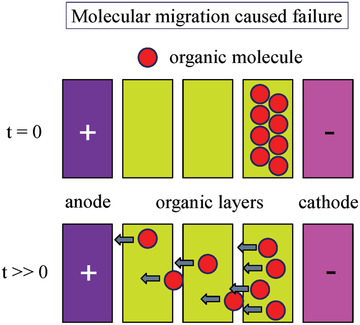
Molecular migration causing OLED device failure.

**Figure 15:**
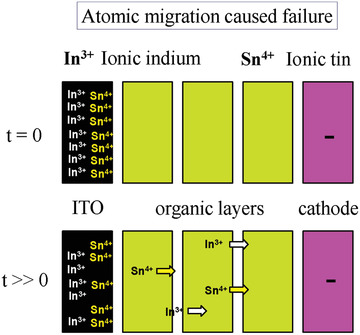
Atomic migration causing OLED device failure.

In OLEDs, due to the continuous flow, electrons collide each other, which leads to molecular migration, resulting in the loss of device efficiency and a decrease in lifetime.^[^ ^134^, ^135^ ^]^ According to Shen et al. mobile ions induced voltage changes, leading to device degradation.^[^ ^136^ ^]^ According to Probst et al., if the metal/organic layer interaction is feeble in the interface, it will enhance the metal migration.^[^ ^137^ ^]^ Lee et al. reported that the presence of Indium in the organic layer increased the driving voltage and reduced luminance efficacy.^[^ ^138^ ^]^ Lin et al. reported that the small organic molecules like TPBi can start migration even at a low voltage bias.^[^ ^139^ ^]^ Png et al. observed the PEDOT^+^ accumulation at the interface between poly(3,4‐ethylenedioxythiophene) polystyrene sulfonate (PEDOT:PSS) and hole injection layer (HIL) even low electric field 1 V cm^−1^.^[^ ^140^ ^]^ Ke et al. and Chua et al. introduced a parylene layer at the electrode–organic interface, in order to prevent atomic migration, the device showed better luminance efficacy and lifetime.^[^ ^141^ ^]^ The molecular and atomic migration failure shown in **Figures** **14** and **15**.

#### 2.2.5 Photochemical Degradation

Organic and polymeric materials are susceptible to various photo‐ and electrochemical reactions in the device.^[^ ^142^, ^143^, ^144^ ^]^ Scholz et al. propose that the photochemical reactions mainly occur at ITO/organic interface in three key steps; light absorption by the polymer layer, the exciton movement toward ITO, and the formation and release of oxygen from the ITO surface.^[^ ^142^ ^]^ He also studied the effect of light‐induced dimerization of ETL materials, namely BPhen, BCP, and Alq_3_.^[^ ^142^ ^]^ In 1996, Rothberg et al. reported the significant role of excitons in OLED device degradation.^[^ ^145^ ^]^ In 2017, Bell et al. describe the exciton induced intramolecular cyclization of arylamine HTL moieties.^[^ ^146^ ^]^ Wang et al. reported that the interaction between HTM positive polarons and singlet excitons increases the hole transport material/electron transport material (HTM/ETM) interface degradation.^[^ ^147^ ^]^ The UV irradiation enhances the device degradation by the formation of singlet excitons (Section 2.2.3). According to Meerheim et al., incorporation of a hole blocking layer plays a significant role in controlling exciton distribution.^[^ ^148^ ^]^ Laser‐desorption/ionization time‐of‐flight mass spectrometry (LDI‐TOF‐MS) is a highly efficient technique to understand the mechanism of photochemical reaction in the device.^[^ ^149^ ^]^

Photochemical degradations are happening due to the irradiation of UV light also. According to Patel et al., continuous exposure of UV light on OLED devices would lead to the formation of dim or dark spots.^[^ ^150^ ^]^ Kondakov et al. found, under UV irradiation, CBP (*N*,*N*′‐dicarbazolyl‐4,4′‐biphenyl) underwent an exocyclic C—N bond cleavage.^[^ ^151^ ^]^ In 2017, Yu et al. reported that the PL quantum yield of CBP decreases 80% after irradiation of UV for 72 h. They attributed that to the morphological changes associated with UV exposure.^[^ ^152^ ^]^ Quirino et al. reported that the decrease in PL intensity was directly proportional to the UV irradiation time, based on an Eu‐*β*‐diketonate complex (Eu(btfa)3bipy) containing device.^[^ ^153^ ^]^ According to Seifert et al., UV irradiation and electrical aging showed the same undesirable impact on device lifetime and the singlet excitons formed by UV exposure played an important role in device degradation.^[^ ^154^ ^]^ By reducing the undesirable UV irradiation, one can control the formation of singlet excitons and the dissociation of weak bonds.

Besides, photochemical degradation, exciton induced degradation mechanism also play a significant role in the device degradation. In 2018, Kim et al. explained the charge neutral generation of polaron pairs by electron transfer from dopant to host excitons.^[^ ^155^ ^]^Aziz et al. reported exciton diffusion the generation of quenchers in HTL and diffusion of excitons from HTL to EML play a crucial role in the device degradation.^[^ ^156^ ^]^ Hany Aziz et al. is one of the pioneers in the field of OLED degradation studies. Recently, his team investigated the exciton‐induced degradation of HTLs and the influence on the efficiency and lifetime of phosphorescent OLEDs.^[^ ^158^ ^]^ Exciton stress on HTLs may reduce the EQE and lifetime of PhOLEDs. Besides, they examined the influence of excitons and electrons in PEDOT:PSS (HIL) degradation.^[^ ^157^ ^]^ They conclude that the incorporation of HTL with PEDOT:PSS HIL can reduce the electron leakage to HIL and improves device stability. His team compared the morphological properties of spin‐coated and blade coated organic semiconductor films.^[^ ^158^ ^]^ The device with the blade coated HTLs and EMLs were less aggregated and showed better efficiency and lifetime. They studied the exciton induced molecular aggregation in organic small‐molecule electroluminescent materials, which showed solution‐processed films are more susceptible to exciton induced aggregation compared to vacuum deposited thin film.^[^ ^159^ ^]^ They reported the influence of the deposition rate on the thin film morphology and PhOLED EL performance.^[^ ^160^ ^]^ Lower deposition rate can reduce the exciton induced degradation and thereby improving the morphological order and stability.

#### 2.2.6 Electrochemical Degradation

Irreversible chemical/electrochemical reactions show some profound impact on the degradation of OLEDs.^[^ ^149^ ^]^ According to Xia et al., the design of electrochemically stable EML is critical for long lifetime OLEDs because the EML contains both electron and hole charge carriers.^[^ ^161^ ^]^ Aziz et al. reported the electrochemical reactions between the electrodes lead to both microstructural change and corrosion. In addition, the presence of short circuit point accelerates the electrochemical degradation process.^[^ ^129^ ^]^ The same group also reported that the anodic oxidation of tris(8‐hydroxyquinoline)aluminum (Alq_3_) and instability of Alq_3_ cations play an important role in device degradation.^[^ ^162^ ^]^ According to Ke et al.,^[^ ^74^ ^]^ Franky So et al.,^[^ ^92^ ^]^ and Gardonio et al.^[^ ^163^ ^]^ due to the presence of moisture at metal/organic interface the electrochemical processes in OLEDs lead to the formation of “bubbles” probably filled with H_2_ gas and hence form dark spot. Savvateev et al. reported that the local current densities formed around the bubble's perimeter would cause to the formation of local luminescence and heating, preventing uniform current injection to the entire device.^[^ ^164^ ^]^ The merging of bubbles also reduces the available injection area. Avoidance of moisture by different encapsulation techniques (Section 2.2.2) is a most effective approach to prevent the electrochemical degradations. Rudmann et al. suggested that more electrochemically stable metals like Ag be used as the cathode to increase device lifetime.^[^ ^165^ ^]^

#### 2.2.7 Electric Breakdown

The term electric breakdown is mainly associated with the dielectric breakdown of OLED materials. Very few reports are available on the dielectric properties of OLED materials. According to Wang et al. the device efficiency and brightness can improve with an electron injection layer with high dielectric strength.^[^ ^166^ ^]^ Ohta et al. examined the effect of dielectric strength of materials in active matrix OLEDs.^[^ ^167^ ^]^ In 2018, Jou et al. reported that the dielectric breakdown of organic materials is directly related to the roughness of the substrate using.^[^ ^168^ ^]^ Even though the driving voltage of OLED devices are 3–5 V, for higher brightness the device requires a higher voltage. After reaching 10 V or above, luminance starts to decrease. This failure may have attributed to the dielectric breakdown of organic materials.

#### 2.2.8 Thermomechanical Failures

In OLED devices, each layer of different materials has its own thermal expansion coefficient, which generates an intrinsic stress.^[^ ^132^, ^169^ ^]^ The stress formed in the layers is released through delamination.^[^ ^132^ ^]^ According to Lee et al., OLED devices are exposed to mechanical loading like bend, torsion, and folds which leads to delamination.^[^ ^132^ ^]^ The thermomechanical properties of materials will strongly influence the flexible OLEDs lifetime and performance.^[^ ^170^ ^]^ Brand et al. suggested that it is necessary to match the coefficient of thermal expansion, elastic moduli and adhesion strength of the secondary material in the devices.^[^ ^171^ ^]^ In 2017, Hasegawa et al. introduced a super heat resistant polymer poly(benzoxazoleimide)s (PBOI) substrate with a very low coefficient of thermal expansion and adequate ductility for flexible OLED devices.^[^ ^172^ ^]^ Ryu et al. fabricated glass fiber reinforced transparent composite films with good thermal conductivity, low coefficient of thermal expansion and high mechanical properties.^[^ ^173^ ^]^ Oh et al. reported that due to the low coefficient of thermal expansion (13 ppm °C^−1^) polyethylenenaphthalate (PEN) showed better performance in flexible OLEDs.^[^ ^174^ ^]^ According to Behrendt et al. the Al_2_O_3_/TiO_2_ nanolaminates grown by atomic layer deposition (ALD) generated a low intrinsic tensile strength. In order to achieve long lifetime OLEDs, it is essential to select the materials with excellent thermomechanical properties.

#### 2.2.9 Thermal Breakdown/Degradation

**Figure 16:**
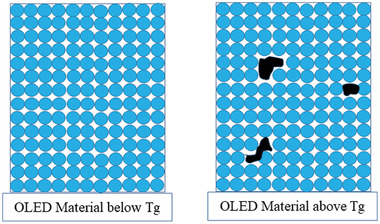
General schematic illustration of the influence of glass transition temperature (*T*_g_) in the thermal degradation of OLED materials.

Thermal instability of the materials used in OLEDs might cause irreversible device degradation.^[^ ^93^ ^]^ Nenna et al. reported that electrical failure mechanism in archetypal OLEDs is mainly related to the lower glass transition temperature of the material, which also restricted the device operating voltage.^[^ ^175^ ^]^ Using X‐ray specular reflectivity, Fenter et al. reported that large thermal expansion behavior of (*N*,*N*′‐diphenyl‐*N*,*N*′‐bis(3‐methylphenyl)‐1,1′‐biphenyl)‐4,4′‐diamine (TPD) to be attributable to its low glass transition temperature, suggesting a strain‐driven failure mechanism.^[^ ^176^ ^]^ Kwak et al. reported that the real‐time temperature of the device with the structure of ITO/*α*‐NPD/Alq_3_/TPBi/LiF/Al is much lower than that of the device without the ETL and the electron injection layer (EIL). The insertion of the suitable ETL and EIL lowers the junction potential barrier and thus advances the efficiency of the electron injection.^[^ ^177^ ^]^ By using transmission matrix model, Bergemann et al. found that the internal air gap between the package lid and substrate provides a high impedance to heat transfer, and eliminating the gap facilitates heat transfer and allows the device to operate at near ambient temperature even at high brightness.^[^ ^178^ ^]^ According to Zhou et al. electricfield induced decomposition of ITO and electro‐migration of indium are responsible for the catastrophic failure or thermal breakdown of OLEDs.^[^ ^179^ ^]^ Morphological changes in the NPB (*N*,*N*‐di(naphthalene‐1‐yl)‐*N*,*N*‐diphthalbenzidine) and Alq_3_ layers at elevated temperature leading to deterioration in the current–voltage characteristics were reported by Xu et al.^[^ ^180^ ^]^ The materials degradation above *T*_g_ is shown in **Figure** **16**. Above *T*_g_, the material is showing degradation, which depicted by black portions in Figure 16.

Joule heat causes thermal expansion, inter‐layer diffusion and crystallization or melting of organic materials, which limits the lifespan of devices.^[^ ^69^, ^181^, ^182^, ^183^ ^]^ Gärditz et al. reported that the rise in device temperature increases local current flow, leading to brightness inhomogeneity in OLEDs.^[^ ^181^ ^]^ Tyagi et al. attributed the Joule heat to the interfacial resistance existing between the organic/organic and metal/organic interfaces.^[^ ^184^ ^]^ Gong et al. used scanning tunneling microscopy (STM) and photoluminescence (PL) to investigate the phase separation, interfacial structural change, and aggregation of BT (1,4‐bis(benzothiazole‐vinyl) benzene) and TPBi (2,2′,2″‐(1,3,5‐Benzinetriyl)‐tris(1‐phenyl‐1‐*H*‐benzimidazole)) caused by joule heat.^[^ ^183^ ^]^ Fujihira et al. reported that high localized current in devices would increase the joule heat.^[^ ^77^ ^]^ Liao et al. observed bubbles to form within OLED devices that may be due to the release of gases caused by high joule heat generated at localized electrical shorts.^[^ ^185^ ^]^

**Figure 17:**
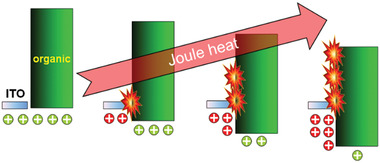
High injection barrier generating high joule heat.

Several methods had been recommended to reduce the effect of joule heat and hence improve the device performance. Prevention methods for Joule heat are the utilization of substrates or anode with high thermal conductivity, employing graded mixed layer of ETL and HTL, reduction of driving voltage, etc. According to Chung et al.*_,_* substrates with high thermal conductivity could enhance the device performance by conducting the joule heat into the substrate.^[^ ^186^ ^]^ Kim et al. reported the use of a highly conductive anode, G‐PEDOT (an aqueous dispersion of PEDOT:PSS with glycerol), to prevent the negative impact of joule heat.^[^ ^187^ ^]^ Tyagi et al. reported the insertion of F_4_‐TCNQ (2,3,5,6‐tetrafluoro‐7,7′,8,8′‐tetra cyano quino dimethane) between the anode/hole transport layers to reduce the interface resistance and joule heat.^[^ ^188^ ^]^ Chwang et al. introduced the graded mixed layer (uniformly mixed ETL and HTL) to diminish the joule heat by reducing the electricfield across the layers.^[^ ^189^, ^190^ ^]^ Matsushima et al. reported that the reduction of driving voltage can enhance the lifetime of OLEDs by preventing the joule heat.^[^ ^189^ ^]^ In 2014, Park et al. introduced films of heat sink in order to dissipate the heat generated by the organic layers in OLEDs.^[^ ^191^ ^]^ Joule heat generation is demonstrated in **Figure** **17**.

Recently, Reineke et al. reported an experimental proof of Joule heating‐induced switched‐back regions in OLED devices.^[^ ^192^ ^]^ Zojer et al. explained how the thermal conductivities of organic layers determine the temperature inside the device.^[^ ^193^ ^]^ Schwamb et al. investigated the passive cooling of lagre area OLEDs, mainly focused on convective cooling.^[^ ^194^ ^]^ Zakhidov et al. introduced hydroflouroethers for heat dissipation in OLEDs.^[^ ^195^ ^]^ Fischer et al. demonstrated electric bistability produced by self‐heating onto the thermally activated conductivity.^[^ ^196^ ^]^ In another report, Fischer et al. described temperature‐activated transport in organic semiconductors and the catastrophic failure associated with that.^[^ ^197^ ^]^

#### 2.2.10 Presence of Impurities

**Figure 18:**
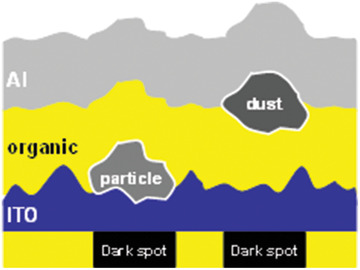
External dust particles causing formation of dark spots.

The efficiency and lifetime of OLEDs are significantly influenced by the presence of impurities and surface roughness. Vardeny et al. and Zou et al. reported the effect of material purity on device performance.^[^ ^198^, ^199^ ^]^ According to their finding, high material purity enhances the luminous efficacy and carrier injection conditions by reducing both ionic diffusion and internal electric field formation. Yamawaki et al. reported the halogenated impurities in OLEDs to trigger radical formation, which can react with the organic materials either by excitation or reduction and cause damage to ETL, EIL, and EML.^[^ ^200^ ^]^ Yamawaki et al. and Becker et al. suggested that the presence of halogenated impurities could be estimated using high performance liquid chromatography–mass spectroscopy (HPLC‐MS) and combustion ion chromatography (CIC).^[^ ^200^, ^201^ ^]^ Presence of dust particles increase the formation of pinholes in the cathode, which leads to infiltration of moisture and air into the device.^[^ ^81^ ^]^ The dust particle penetration is shown is **Figure** **18**.

Adachi et al reported some studies related to the influence of impurities in device performance. According to him, if the vacuum chamber cleaned by plasma cleaning and the partial pressure of the water reduces by a cryotrap, device lifetime will be stable.^[^ ^202^ ^]^ In another study, his team reported that impurities floating inside the vacuum chamber seriously influencing the lifetime value and reproducibility of the device.^[^ ^72^ ^]^ They reported that the device fabricated in a new chamber can attain a lifetime approximately twice than that of a pre‐existing chamber.^[^ ^203^ ^]^

Several methods are attributed to identify and reduce the impurity content associated with OLED fabrication . Tominetti et al. reported an efficient mass spectrometric technique for detecting internal impurities in OLED displays.^[^ ^204^ ^]^ Tsugita et al. fabricated high purity organic films via gas flow deposition method (GFD).^[^ ^205^ ^]^ According to Jain et al.,^[^ ^206^ ^]^ Salati et al.,^[^ ^207^ ^]^ and Pardo et al.^[^ ^208^ ^]^ recrystallization and vacuum deposition are efficient methods to purify both organometallic and organic compounds used in OLEDs.

Various surface treatments were introduced to tune the surface roughness of ITO.^[^ ^209^ ^]^ According to Li et al. and Jung et al. the surface roughness can be decreased by etching (acid/alkali), annealing, mechanical polishing, etc.^[^ ^209^, ^210^ ^]^ Choi et al. reported boron doping affects the surface properties of ITO but increases the OLED performance because it influences surface energy, transmittance, sheet resistance, work function and mobility.^[^ ^211^ ^]^ Zhou et al. examined the sand blasting technique for creating surface roughness on OLED substrate, in order to reduce the wave nature of internally generated photons, which could increase the external quantum efficiency from 9% to 11.6%.^[^ ^212^ ^]^ Hatton et al. reported that Silane modified ITO shows better power efficiency as compared to normal ITO surface due to tune the anode work function to the HOMO of HTL.^[^ ^213^ ^]^ Kim et al. investigated the peak‐to‐valley roughness (*R*_pv_) of ITO surface directly proportional to the leakage current of OLED.^[^ ^214^ ^]^ Park et al.^[^ ^215^ ^]^ and Lu et al.^[^ ^215^ ^]^ observed plasma treatment reduces both surface roughness and contaminations. Helander et al. reported the chlorination of transparent ITO improved the electrode work function (>6.1 eV).^[^ ^216^ ^]^

## 3 Materials for Long Lifetime OLED

The performance and lifetime of OLEDs primarily depend upon the characteristics of the hole transporting materials (HTM), electron transporting materials (ETM), emissive materials (EM), and host materials.^[^ ^1^, ^3^, ^7^ ^]^ Fluorescence, phosphorescence, and thermally activated delayed fluorescence (TADF) are the main mechanisms associated with the emission phenomenon in OLEDs.^[^ ^7^ ^]^ Fluorescent materials emit light only due to the consumption of singlet excitons, while phosphorescent materials can utilize both singlet and triplet excitons.^[^ ^217^ ^]^ TADF materials utilize both singlet and triplet excitons by converting triplet excitons to singlet excitons through reverse intersystem crossing.^[^ ^218^ ^]^ Aggregation‐induced emission (AIE) is an interesting phenomenon where molecules which are otherwise nonemissive in the solution state are found to emit strongly in the aggregate form or solid state.^[^ ^219^ ^]^ A high energy transfers between the host and guest results in yielding a longer lifetime for the OLED device.^[^ ^220^ ^]^ Numerous research studies and key publications proposed that through reduction of host singlet‐triplet splitting, precise adjustment of host–guest energy gap and doping concentration, and enhancement of host emission‐guest absorption overlapping a long operational lifetime TADF and TADF‐sensitized OLED device can be fabricating, even higher than PhOLEDs.^[^ ^221^ ^]^

**Figure 19:**
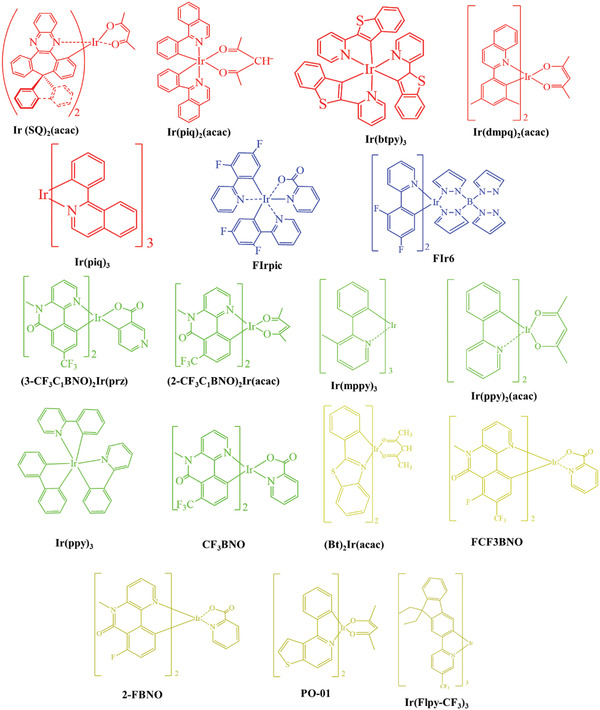
Molecular structures of heavy metal complexes based phosphorescent emitters.

**Figure 20:**
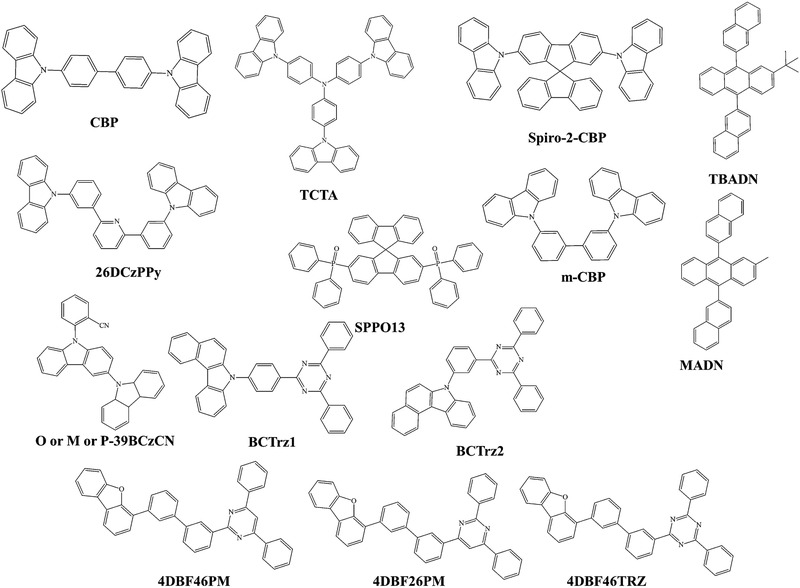
Molecular structures of host materials using in both fluorescent and phosphorescent OLEDs.

**Figure 21:**
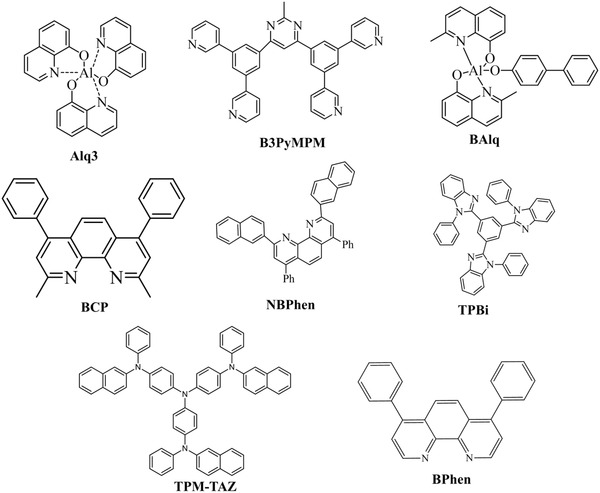
Molecular structures of ETL materials using in OLEDs.

Many research groups are working on the research and development of blue fluorescent materials. Naphthalimide, pyrene, phosphine oxide, oxidazoles, benzo‐fluoranthene, and phenanthroimidazole materials are extensively using as blue emitters.^[^ ^222^ ^]^ In 2018, Jung et al. reported a pyre based blue emitter, which showed the LT95 lifetime of 471 h and extrapolated LT_50_ lifetime of 30000 h.^[^ ^223^ ^]^ A wide range of phosphorescent emitters have been reported in the past few years, most of them being Iridium based, as shown in **Figure** **19**. Host materials play a major role in host–guest energy transfer and exciton confinement.^[^ ^224^ ^]^ **Figure** **20**. shows the range of host materials. **Figure** **21** depicts the range of ETL materials commonly using in OLEDs.

**Figure 22:**
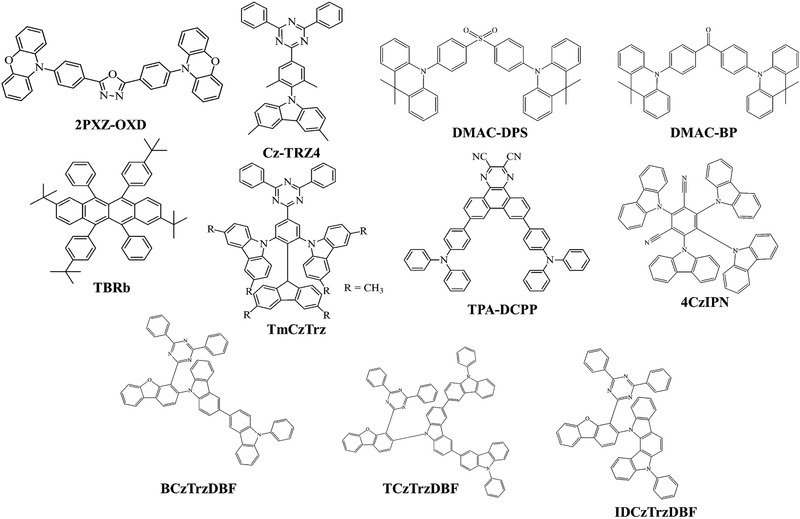
Molecular structures of TADF emitters using in OLEDs.

**Figure 23:**
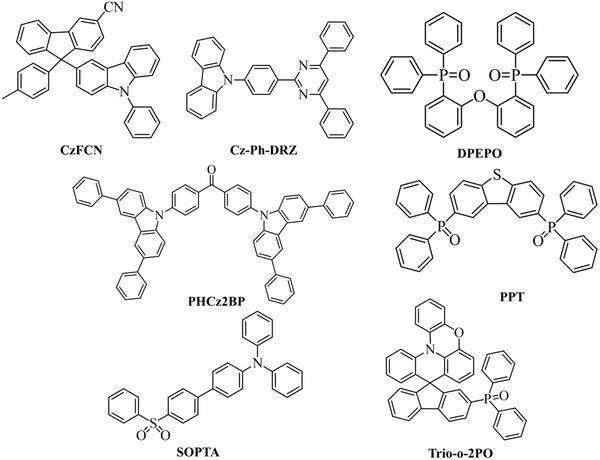
Molecular structures of TADF host materials using in OLEDs.

Organic small molecules, polymers and dendrimers are widely employed as TADF materials.^[^ ^225^ ^]^ Recently, Lee et al. reported an in‐depth review of TADF materials which find use in the fabrication of long lifetime OLEDs.^[^ ^226^ ^]^ They proposed an efficient device lifetime expansion strategy for the TADF OLEDs in terms of the rational chemical bond stabilizing molecule design and fast reversed intersystem crossing rate model. According to Adachi et al. n‐type hosts are appropriate for stable electroluminescent devices because of their capability to balance charge fluxes and suppress high‐energy exciton formation.^[^ ^227^ ^]^ Liao et al., pure hydrocarbon hosts which possess high glass transition temperature making them suitable for being used as OLED materials.^[^ ^228^ ^]^ Green TADF OLEDs with SF3‐TRZ (2‐(9,9′‐spirobi[fluoren]‐3‐yl)‐4,6‐diphenyl‐1,3,5‐triazine (SF3‐TRZ)) as the host were seen to achieve a maximum EQE of 20.6% and *T*_50_ of 10934 h for an initial brightness of 1000 cd m^−2^. More importantly, SF3‐TRZ can also function as a host for sky‐blue TADF OLEDs because of its high T_1_. A sky‐blue TADF OLED with a high EQE of 8.8% and a lifetime of 454 h for an initial brightness of 1000 cd m^−2^ was produced.^[^ ^229^ ^]^ Some frequently using TADF emitters and host materials are shown in **Figures** **22** and **23**.

TADF and hyperfluorescent based OLED devices demonstrating better quantum efficiency compared to the phosphorescent and fluorescent counterparts.^[^ ^230^, ^231^, ^232^, ^233^ ^]^ The operational lifetime of TADF OLEDs is much lower compared to those of phosphorescent OLEDs.^[^ ^234^ ^]^ Many research groups are presently investigating avenues to improve device performance through effective utilization and crucial device engineering to improve the potential ability of TADF materials. Sasabe et al. proposed a lifetime extending strategy of thermally activated delayed fluorescent OLEDs from a molecular design approach that significantly improved device efficiency and lifetime.^[^ ^235^ ^]^ A novel sterically bulky hexaphenylbenzene based HTMs (TATT, 4DBTHPB, 4DBFHPB), 4CzIPN based device showed an EQE of 19.4% and LT_50_ of 24 000 h.

According to Lee et al., molecules with chemical bond stabilizing design and high reverse intersystem crossing effect can improve the lifetime of TADF OLEDs.^[^ ^234^ ^]^ Yu et al. improved the efficiency and lifetime of TADF OLED by the utilization of novel emitters (BCzTrzDBF, TCzTrzDBF, IDCzTrzDBF) via replacing common phenyl linker with dibenzofuran linker.^[^ ^236^ ^]^Recently, Jeon et al. published a very detailed progress report about TADF OLED lifetime improvement for the last seven years.^[^ ^230^ ^]^Jang et al. enhanced the TADF device by the utilization of electrostatic potential dispersing pyrimidine‐5‐carbonitrile acceptor.^[^ ^237^ ^]^Kido et al. improved the TADF OLED lifetime by developing novel hexaphenylbenzene‐based sterically bulky hole transporters.^[^ ^238^ ^]^

According to Yang et al., utilization of both charge‐transfer exciton (CT) and local exciton (LE) is an efficient pathway for obtaining high efficiency devices.^[^ ^239^ ^]^ They reported hybridized local and charge‐transfer excited state (HLCT) mechanism can utilize for the fabrication of highly efficient OLEDs.^[^ ^240^ ^]^ Ma et al. reported a “hot exciton” reverse intersystem crossing (RISC) process from T2 to S1, which evade the accumulation of long‐lived triplet excitons and the nondoped device showed a maximum EQE of 10.5%.^[^ ^241^ ^]^ In another work, they reported a high radiative exciton ratio of 48% (exceeds limit of 25% in conventional fluorescent OLEDs), which may due to the result of efficient RISC through the hot‐exciton process.^[^ ^242^ ^]^

Still, the OLED industry is facing challenges related to the stability of blue OLED. Various approaches are adopting to improve the efficiency and lifetime of blue OLEDs. Recently, Lin et al. published an informative review paper on current status, challenges, and future outlook of blue organic light emitting diodes.^[^ ^43^ ^]^ According to them, blue phosphorescent and TADF OLEDs are satisfactory with the efficiency and emission color but not achieving a reliable lifetime. Even though the limited efficiency, blue fluorescent, and triplet‐triplet florescent (TTF) OLEDs are widely using for display applications.

Recently, Yang et al. examined the key factors affecting the lifetime of blue phosphorescent OLED using CN modified blue host materials (O‐39BCzCN, M‐39BCzCN, P‐39BCzCN).^[^ ^243^ ^]^ He concluded that bond dissociation energy and Forster energy transfer rate are crucial to the lifetime of blue phosphorescent OLEDs. Park et al. reported a long lifetime red PhOLED by the utilization of benzocarbazole and diphenyltriazine based bipolar host materials (BCTrz1, BCTrz2). The device lifetime increased 30 times compared to the bipolar host material, CBP.^[^ ^244^ ^]^ Kido's group improved the red PhOLED lifetime by the introduction of a novel dibenzofuran‐based n‐type exciplex host (4DBF46PM, 4DBF26PM, 4DBF46TRZ).^[^ ^245^ ^]^ The device showed an LT_80_ of 3300 h, which is six times longer than the previously reported lifetime for deep‐red OLED.^[^ ^246^ ^]^ Yamazaki et al. introduced a novel host–guest system that improves the lifetime of a deep‐red phosphorescent OLED ≈5.4 compared to the conventional system.^[^ ^247^ ^]^

The effective utilization of suitable materials in the exciplex forming cohost system improving the lifetime of OLED devices. Recently, Lee et al. reported a p‐type host ((3,3′‐bis(5‐pheny‐ lindolo[3,2‐*a*]carbazol‐12(5*H*)‐yl)‐1,1′‐biphenyl (IDCzBP)) for an exciplex host, which improved the device lifetime of green PhOLED by 11 times compared to the conventional p‐type host system.^[^ ^248^ ^]^ Yamaguchi et al. reported a high temperature (85 °C) withstanding deep red PhOLED with exciplex forming host and guest material.^[^ ^249^ ^]^ Kim et al. synthesized high triplet energy exciplex hosts triphenylsilyl blocking groups, which showed device lifetime of LT_80_ of 1900 h at 100 cd m^−2^ and 21.6% EQE.^[^ ^250^ ^]^ Recently Lee et al. published an informative review paper describing exciplex hosts for blue phosphorescent OLEDs.^[^ ^251^ ^]^ Wong et al. reported an exciplex forming cohost with biscarbazole donor and a triazine‐based acceptor to improve the efficiency and lifetime of fluorescent and phosphorescent OLEDs.^[^ ^252^ ^]^ Song et al. examined the performance RGBY OLEDs by employing an electroplex host system. The devices showed a longer lifetime compared to a single host, mixed host, and exciplex host devices.^[^ ^253^ ^]^

**Figure 24:**
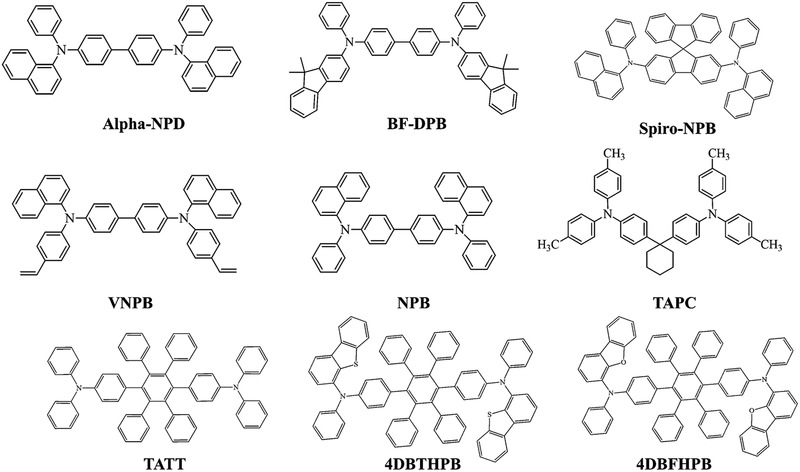
Molecular structures of HTL materials using in OLEDs.

Charge transporting materials have the capability to accept the charge from the electrode and thereafter inject the carriers into the emissive zone. Molecular architecture, thermal stability, morphology, highest occupied molecular orbital (HOMO)–lowest unoccupied molecular orbital (LUMO) values, ability to block the carriers of opposite charge and charge mobility of charge transporting materials are essential for efficient and long lifetime OLEDs. Hole transporting materials (HTM) possess low ionization potential and low electron affinity, whilst electron transporting materials (ETM) possess high ionization potential and high electron affinity.^[^ ^1^, ^254^ ^]^ The glass transition temperature (*T*_g_) of both HTM and ETM strongly influences the lifetime of OLEDs.^[^ ^255^ ^]^ Triarylamine based hole transporting materials have low glass transition temperature compared to carbazole‐based materials.^[^ ^256^ ^]^ Thermal breakdown of OLEDs has been discussed in Section 2.2.9. Silane moieties also function as hole transporting materials. Some other widely using hole transporting materials have been shown in **Figure** **24**. Oxidazole molecules, dendrimers, metal chelates, azole‐based materials, etc., are commonly using as electron transport materials.^[^ ^254^ ^]^ Thermal evaporated devices showed a better lifetime than solution‐processed devices. Small molecules and polymeric materials are generally used as carrier transporting materials in OLEDs.

## 4 Device Architecture for Long Lifetime OLED

Device degradation which is quantified by the lifetime of the devices is an efficiency loss process that happens in the OLEDs overtime during the electrical driving process, which originated from the inherently poor stability of organic photochromic materials inserted in the OLEDs. Numerous degradation processes of OLEDs have their origin in the improper management of device structures, which aggravate material degradation. Therefore, both material and device‐related degradation routes should be managed at the same time. Device architecture is considered to be an imperative factor that influences the device efficiency, performance and lifetime.^[^ ^164^, ^257^, ^260^ ^]^ Various OLED devices designed approach for long‐lifetime is discussed below in detail.

### 4.1 Stepwise Devices

A high operating voltage and short operation lifetime are the major concerns of the current OLED devices in applications as compared to the other competitive display technologies. In order to break through these limitations, organic electroluminescent materials holding high conductivity and good electrical relation with electrodes are desired. Stepwise, double emissive layer, novel carrier/charge transport materials, and metal‐doped complex based structural approach are extensively employed to enhance the device lifetime. Recently, doping techniques, which have been widely utilized in forming inorganic semiconductors, have been pertained for shaping organic p‐ and n‐type materials.^[^ ^258^, ^259^ ^]^ In 2005, Lee et al. demonstrated high‐performance OLED devices based on the novel metal doped electron transporting and cathode materials result in the drop of 2.59 V in driving voltage, a 47.3% rise in current efficiency and a 3.14 times improvement in operation lifetime.^[^ ^260^ ^]^ Because of the high thermal stability, better charge balance and good energy alignment of the electron transport layer with the emissive layer the lifetime of the devices significantly enhanced. In 2006, Tsai et al. fabricated a long lifetime and high efficiency white OLEDs with a mixed host in one of the double emission layers. The device exhibited the half lifetime of ≈100 h at initial 5000 cd m^−2^, five times that better than that of the NPB counterpart.^[^ ^261^ ^]^

**Figure 25:**
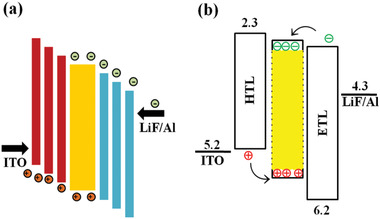
Device architectures of step‐wise energy transfer in OLEDs.

In 2011, Duan et al. presented a strategy to achieve a white organic light‐emitting diode (WOLED) with an extremely long lifetime through the wise control of the recombination zone. They achieved a record high lifetime of over 150 000 h at an initial brightness of 1000 cd m^−2^, ≈40 times longer than the conventional bilayer WOLED.^[^ ^262^ ^]^ Device composed of a blue emissive layer of 6,6′‐(1,2‐ethenediyl)bis‐*N*‐2‐naphthalenyl‐*N*‐phenyl‐2‐naphthalenamine (ENPN) doped in 9‐(1‐naphthyl)‐10‐(2‐naphthyl)‐anthracene (*α*,*β*‐ADN) was deposited on top of the mixed host blue emissive layer to prevent hole penetration inside the electron transport layer and to achieve better confinement of carrier recombination. Therefore, the employment of double emitting layers can stabilize the blue emission, which is the key feature to their device performance. In order to expand the operating lifetime, the uniform or graded mixed host structure has been initiated to eliminate the sharp heterojunction interface.^[^ ^190^, ^263^, ^264^ ^]^ In 2012, Yang et al. demonstrate an excellent phosphorescent green organic light emitting diodes with double emitting layer and lithium fluoride (LiF) doped 2,2′,2″‐(1,3,5‐Benzinetriyl)‐tris(1‐phenyl‐1‐*H*‐benzimidazole) (TPBi) as an electron transporting layer. The device exhibits a current efficiency of 40.5 cd A^−1^, power efficiency of 23.7 lm W^−1^ and operational lifetime of 5300 h, nearly 1.99, 2.95, and 35 times of those of the reference device, respectively.^[^ ^265^ ^]^ The reason for the significant improvement is the effective carriers self‐balancing character of double emitting layer OLEDs, less numbers of heterojunction interface and superior electron transport property of TPBi:LiF. In the same year, Nakayama group developed a phosphorescent white OLED device which exhibits a low drive voltage of 3.5 V, a power efficiency of 64 lm W^−1^, EQE of 20%, and a lifetime of 10 000 h at an initial luminance of 1000 cd m^−2^ with a light out‐coupling technique.^[^ ^266^ ^]^ **Figure** **25**. Shows the device architectures of step‐wise energy transfer in OLEDs.

In 2013, Hao and co‐workers demonstrated a highly efficient and long working lifetime phosphorescent organic light emitting diode containing mixed host composed of wide‐bandgap based 4,7‐diphenyl‐1,10‐phenanthroline (Bphen) and 4,4ʹ‐bis(carbazol‐9‐yl)‐biphenyl (CBP).^[^ ^267^ ^]^ The mixed host based devices exhibited an operation lifetime of 3530 h at a luminance of 500 cd m^−2^ intensifying by about 4.1 and 2.4 times relative to that of the counter single host and double emitting layer devices. High‐efficiency and longer working lifetime were credited to the absence of heterojunction and balanced charge carrier transport characteristics in the mixed host based OLED structures. In 2014, Cho et al. investigate a universal host (DCzDCN) material for both green thermally activated delayed fluorescence and phosphorescent OLEDs and achieved a high quantum efficiency of 25% and long lifetime around 200 h.^[^ ^268^ ^]^ The suppression of exciton‐polaron annihilation has been recognized as an effective way to increase the operational lifetimes of OLEDs. The operational lifetime of an OLED utilizing a TADF emitter was noticeably improved by employing interlayers of an electron injection material and a ten‐fold rise in the lifetime of a blue PhOLED was attained by employing a graded dopant concentration profile in a broadened emitting layer.^[^ ^269^, ^270^ ^]^ More recently, Fukagawa et al. proposed that the stability and lifetime of the phosphorescent devices is well‐nigh proportional to the Förster resonance energy transfer rate from host to emitter when thermally activated delayed fluorescence molecules are used as hosts. The 2c host based devices expressed extremely high operational lifetime LT_50_ of 20 000 h, Δ*E*_ST_ 0.29 eV, and an EQE of 21.5%.^[^ ^220^ ^]^ For prospect, further improvement of lifetime will be necessary while improving power and current efficiency. If a device of longer lifetime is realized, the foot of the application spreads out greatly.

### 4.2 Tandem Devices

A tandem organic light‐emitting diode (OLED) has multiple electroluminescence (EL) units attached electrically in series with unique intermediate connectors within the device. Researchers have examined this new OLED architecture with growing interest and have found that the current efficiency of a tandem OLED containing N EL units (*N* > 1) should be N times that of a conventional OLED comprising only a single EL unit.

As compared with the conventional organic light emitting diodes, tandem OLED devices have received a broad attention owing to their superior current efficiency, power efficiency, luminescence, and operational lifetime. In tandem OLEDs, two or more individual electroluminescence (EL) units are electrically coupled in series with unique connecting stacks which function as a charge generation layer (CGL), where holes and electrons are generated and injected into the adjacent hole transporting and electron transporting layer, respectively. The key element in fabricating a high performance and long lifetime tandem device is the connecting materials stack, which plays a significant role in the electric field distribution, charge generation and charge injection mechanism.^[^ ^3^, ^271^, ^272^ ^]^

The interconnecting layers are usually formed via combining the p‐ and n‐type layers between the emission units. The p‐type layer comprises oxides such as indium tin oxide (ITO), vanadium pentoxide (V_2_O_5_), molybdenum trioxide (MoO_3_) in addition to p‐doped organic hole‐transporting materials. The n‐type layers composed of an electron transporting materials doped with metals such as Li, Cs and Mg or metal complexes. In 2009, Lee and co‐workers developed an efficient interconnecting layer (TR‐E314:Li/LGC101:NPB) via combining the n‐ and p‐type doped layers. The stacked unit devices expressed a maximum current efficiency, EQE and CIE coordinates of 14.7 cd A^−1^, 10.6% and (0.12, 0.22), respectively, under steady current density of 10 mA cm^−2^. The 20% decay lifetime (*t*_80_) of the stacked OLED (366 h) is 4.4 times that of the single‐unit OLEDs.^[^ ^273^ ^]^ It is apparent that the operating lifetime and current efficiency of the OLEDs is enhanced in the presence of the interconnecting layers. For tandem WOLEDs, the main focus is on how to design an effective charge generation layer, since it plays a significant role in ensuring high efficiency and long lifetime.^[^ ^164^ ^]^

**Figure 26:**
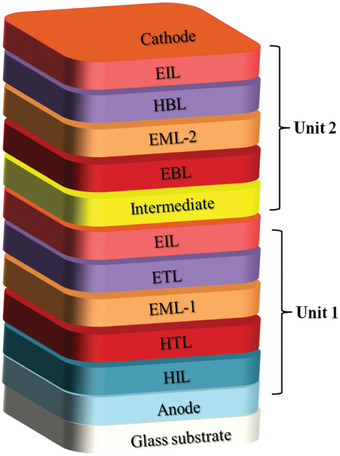
Schematic representation of a two stack tandem OLED device.

In 2013, Hao et al. demonstrate a group of electrophosphorescence organic light emitting devices with different number of heterojunctions. They proposed that the device performance illustrates a gradual enhancement in efficiency and lifetime with the lessening of heterojunction interfaces, which is credited to eliminating effectively the energy barrier between the heterojunction interfaces and the efficient and balanced carrier injection and transport via consuming an appropriate chemical doping of the wide band gap compounds as the charge/carrier transport layers. A single‐heterojunction OLED device showed a maximum PE of 32.1 lm W^−1^, nearly 3.1 times that of the referential multi‐heterojunction PhOLED, and the operational lifetime of 1184 h, beyond 15 times of the reference device.^[^ ^274^ ^]^ The two stack tandem device structure is shown in **Figure** **26**.

A tandem device also permits individual subdevices to be controlled independently, such capability may find numerous fascinating applications of these devices such as color tunable light.^[^ ^275^, ^276^, ^277^ ^]^ To make these devices more efficient Tang et al. fabricated a three‐unit tandem device using a p–n junction as the connecting unit between the electroluminescence units and showed excellent light out coupling and carrier injection characteristics with better operational lifetime.^[^ ^99^ ^]^ One of the key challenges in tandem OLEDs is to facilitate the effective charge generating interconnection between electroluminescence units. To successfully overcome the above problem, each electroluminescence unit is electrically connected in series via a carrier generation layer. In 2016, Fung et al. reviewed the current research advances in tandem OLEDs architecture with key focus on the material selection and interface studies in the intermediate connectors, as well as the optical design of the tandem OLEDs.^[^ ^272^ ^]^ The interface of the intermediate connector is considered as very crucial in determining the driving voltage, current efficiency, power efficiency, and operational lifetime of tandem OLEDs.^[^ ^271^, ^278^ ^]^ In 2017, Song et al. reviewed the recent result of the degradation mechanism, operational stability and lifetime improvement strategies for blue PhOLEDs via classifying them into device and material based approaches.^[^ ^260^ ^]^

Triplet‐triplet annihilation (TTA) and triplet‐polaron annihilation (TPA) are the foremost degradation mechanisms and these arises due to the long excited state lifetime of the triplet excitons. Both the TTA and TPA processes are required in the operation of OLEDs, but they should be minimized to achieve PhOLEDs with long lifetimes. More recently, Shi et al. fabricated a high‐performance hybrid tandem WOLEDs with a thin layer of Ca incorporated in between Liq and HAT‐CN to act as an intermediate connector and achieved the maximum forward‐viewing current efficiency, power efficiency and EQE of 106.3 cd A^−1^, 51.4 lm W^−1^ and 39.6%, respectively.^[^ ^279^ ^]^ In the future, we should still persist to look for other intermediate connectors with negligible voltage drop and high transparency in order to achieve ideal tandem OLEDs with improved voltage stability, current efficiency, power efficiency, power consumption, and operational stability.

### 4.3 p–i–n Devices

**Figure 27:**
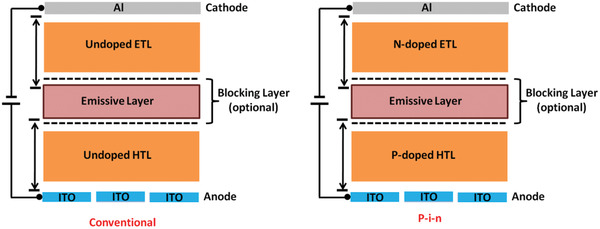
Device architectures of conventional and p–i–n OLEDs.

The high performance and long lifetime demands for OLEDs are met by the p–i–n (p‐doped hole transport layer/intrinsically conductive emission layer/n‐doped electron transport layer) approach. The p–i–n OLEDs demonstrated in **Figure** **27**. The diodes based on p–i–n concept have exponential forward characteristics up to comparatively high current densities. These p–i–n devices enable high luminance and efficiency at extremely low operating voltages, as well as long OLED lifetimes.^[^ ^53^, ^258^, ^259^, ^280^ ^]^ In 2005, Wellmann et al. reported very high‐efficiency devices with power efficiencies >70 lm W^−1^ and lifetimes of more than 220 000 h at a brightness of 150 cd m^−2^ through integrating double emission layer, comprised of two bipolar layers doped with the emitters, into the p–i–n architecture.^[^ ^53^ ^]^

Devices with single emission layers lead to a strong decrease in efficiency at elevated brightness. Improvement of the OLED characteristics is possible when p–i–n architecture was combined with the double emission layer concept, where a predominantly hole transport emission layer and a predominantly electron transport emission layer was doped with the phosphorescent emitter dye. Using the p–i–n layer concept, Meerheim et al. fabricated a very high power efficiency of 37.5 lm W^−1^ and a lifetime of 30 200 h at 100 cd m^−2^ initial luminance OLEDs. He studied the charge carrier and exciton distribution routes for achieving broad and centered exciton recombination zone to maximize the device performance of OLEDs both in terms of lifetime and efficiency.^[^ ^150^ ^]^

In 2008, Fehse et al. investigate the degradation mechanism of p–i–n and i–n–OLEDs on PEDOT:PSS anodes with respect to different anode pretreatments. The extrapolated lifetime difference between indium tin oxide (ITO) and PEDOT:PSS anodes showed 5000 h longer living OLED on a polymer anode.^[^ ^281^ ^]^ Meerheim et al. reviewed the recent advances and improvement strategies concepts in terms of low operating voltages, high power efficiency and long lifetime in the field of p–i–n type organic light emitting diodes (OLEDs).^[^ ^282^ ^]^ In 2012, Birnstock et al. reported a new record lifetime value for p–i–n devices 50% lifetimes of more than 1 million h for fluorescent red, 100 000 h for phosphorescent green based on Ir(ppy)_3_ and 50 000 h for fluorescent blue of starting brightness of 500 cd m^−2^.^[^ ^283^ ^]^ In the same year, Birnstock et al. demonstrated lifetimes of phosphorescent bottom emitting p–i–n OLEDs of 30 000 h at a brightness of 500 cd/m².^[^ ^284^ ^]^ Further progress can be expected from the newly developed molecular n‐dopant and p‐dopant, hence long‐term and thermal stability of p–i–n OLEDs.

### 4.4 Inverted Devices

**Figure 28:**
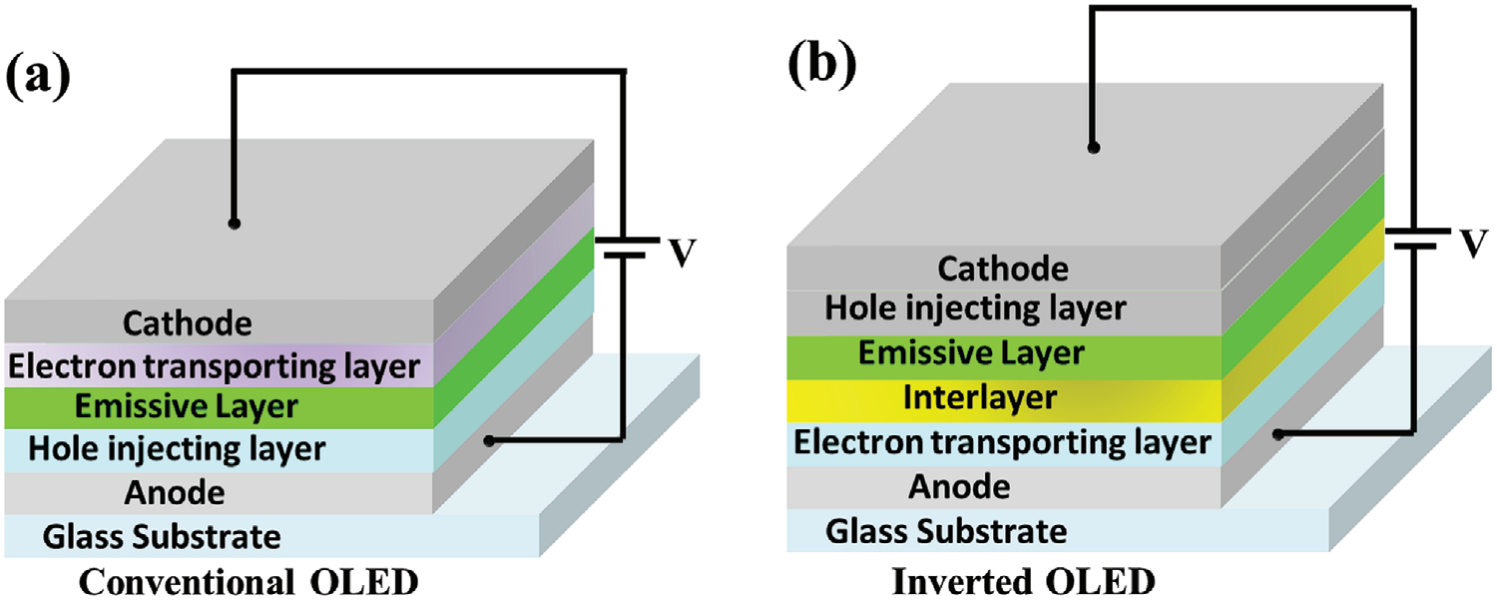
General device structure of a) conventional and (b) inverted OLED device.

OLEDs have been attracting extensive attention due to their excellent optoelectronic properties and superb energy saving features. However, the poor ambient stability remains a critical hurdle for commercialization of this unique technology. In the last few years, inverted OLEDs (iOLEDs) have been proposed as an ideal structure for realizing air‐stable OLEDs because of its several advantages over convention architecture OLEDs. For example, the environmental stability of OLEDs can be significantly improved because reactive materials such as alkali metals which widely used between organic and metal layers in conventional devices replaced with water‐ and oxygen‐sensitive metal oxides electron injection materials such ZnO, SnO_2_, TiO_2_, and ZnO_2_ due to their air stability, nontoxicity, transparency and high electron mobilities.^[^ ^285^, ^286^, ^287^ ^]^ However, iOLED based on these n‐type metal oxides were criticized for lower performance when compared with conventional OLED because there is still a large electron injection barrier from their conduction band to the LUMO of the emitters.^[^ ^288^ ^]^ One of the possible solution these issue is use an interlayer between metal oxide ETL and emissive layer. The general device architecture of conventional and inverted OLEDs are shown in **Figure** **28**.

In 2012, Zou et al. introduced a polymers containing simple aliphatic amine groups PEIE and PEI as a “universal” surface modifiers to reduce the energy barrier between ETL and emissive layer that allow low‐cost environmentally fabrication of air‐stable devices.^[^ ^289^ ^]^ In 2014, Lee et al. demonstrated iPLEDs with a CE of 61.6 cd A^−1^, a PE of 19.4 lm W^−1^ and an EQE of 17.8% by using a simple and effective method that relies on the nanostructure of ZnO‐R and the 2‐ME + EA polar solvent treatment of the ZnO‐R.^[^ ^290^ ^]^ In 2015, they further reported a new interfacial engineering method by introducing a series of amine‐based interfacial molecules that contain 2–6 amine groups (2–6N) for highly efficient iPLED. The best optimized iPLEDs exhibit a maximum luminance of 99 300 cd m^−2^, a CE of 23.1 cd A^−1^, a PE of 8.83 lm W^−1^, and an EQE of 8.40%, which are 30‐, 32‐, 38‐, and 30‐fold higher than that of the reference device without any interlayer.^[^ ^291^ ^]^ Fukagawa et al. reported a long‐lived flexible display using efficient and stable iOLEDs, where ZnO and polyethyleneimine (PEI) were used as ETL and interlayer to effectively inject electron into the emissive zone. in which electrons can be effectively injected without the use of alkali metals. The iOLED‐based flexible display emits light over 1 year under the simplified encapsulation, though the cOLED‐based flexible display shows almost no luminosity only after 21 day under the same encapsulation.^[^ ^292^ ^]^

### 4.5 Exciplex Cohost System Based Devices

**Figure 29:**
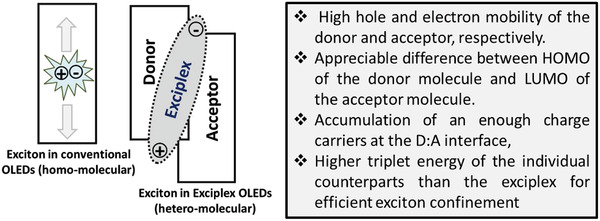
(Left) Schematic representation of excitons in conventional and exciplex OLEDs. (Right) Essential parameters for donor and acceptor to obtain TADF mechanism enabling exciplex forming cohost systems.

OLEDs employing TADF mechanism exciplex cohost systems have gained attractive interest due to the promising low operation voltage, high IQE, low efficiency roll‐off, high light‐out coupling efficiency, and enhanced lifetime. Exciplex‐forming cohost system enables efficient singlet and triplet energy transfers from the host exciplex to the dopant because the singlet and triplet energies of the exciplex are at same level as well as also reduce the probability of direct trapping of charges at the dopant molecules and lower charge‐injection barrier from the charge‐transport layers to the emitting layer. **Figure** **29** showing the schematic representation of excitons in conventional and exciplex OLEDs.

In 2014, Seo et al. reported that the lifetime of OLEDs using exciplex forming cohosts can theoretically reach 1 million h at an initial luminance of 1000 cd m^−2^, as long as the exciplex is used as a medium of energy transfer rather than nonradiative relaxation pathways.^[^ ^293^ ^]^ In 2018, Shih et al. developed an exciplex forming cohost system consisting of a conventional star‐shaped carbazole TCTA and a triazine 3P‐T2T as a donor and an acceptor molecules, respectively. The resultant device exhibited an operational lifetime (*τ*_80_) of ≈1020 min with the initial brightness of 2000 cd m^−2^, which is 56 times longer than the reference device.^[^ ^294^ ^]^ In 2019, Liao's group reported a deep‐red OLED with EQE > 22% employing exciplex cohost system as the main matrix and one TADF molecules as a sensitizer. The lifetime of based optimized device based on exciplex cohost: TADF sensitizer:emitter is approximately twentieth time longer than that of single host based device using a conventional host–guest emitting layer.^[^ ^294^ ^]^ Recently, Wang et al. demonstrated a PQ2Ir based red phosphorescent OLEDs with a maximum EQE of 19.8% and LT_50_ (time to 50% of initial luminance at 1000 cd m^−2^) lifetime up to 10 169 h based on 9,9′‐diphenyl‐9*H*,9′*H*‐3,3′‐bicarbazole (BCzPh):2‐(9,9′‐spirobi[fluoren]‐2‐yl)‐4,6‐diphenyl‐1,3Δ,5‐ triazine (SF2‐TRZ) exciplex cohost system that is ≈16 times longer than the reference group TCTA:2,4,6‐tri [(1,1′‐biphenyl)‐3‐yl]‐1,3,5‐triazine (T2T).^[^ ^295^ ^]^ Some of the unique device structures used for both high efficiency and long lifetime tabulated in **Table** **2**.

**Table 2:** Some unique device architectures/materials for both high efficiency and long lifetime

Device architecture	Color/emission mechanism	EQE [%]	Lifetime [h]/initial luminance [cd m^−2^]	Ref.
ITO/PEDOT:PSS/poly(9‐vinylcarbazole)/SiCz:5CzCN/TSPO1/TPBI /LiF /Al	Blue/TADF	19.7	LT_80_ = 100/500	^[^ ^296^ ^]^
Glass/ITO/NPB:15%MoO*_x_* (40 nm)/NPB (10 nm)/TCTA (10 nm)/EML/TPBi (40 nm)/Liq (1 nm)/Al (100 nm)	Orange/phosphorescent	24.3	LT_50_ = >500/200	^[^ ^297^ ^]^
ITO (50 nm)/BPBPA:HATCN (40 nm, HATCN 30%)/BPBPA (10 nm)/mCBP (10 nm)/mCBP:SiCzTrz (25 nm, 50:50%)/DBFTrz (5 nm)/ZADN (20 nm)/LiF (1.5 nm)/Al (200 nm)	Blue/phosphorescent	27.6	LT_50_ = > 10 000/100	^[^ ^298^ ^]^
ITO(100 nm)/HAT‐CN(3 nm)/ NPB‐ (27 nm)/NPB+BANE(16 nm) /NPB+BANE(19 nm)/Alq_3_ (23 nm)/LiF (1 nm)/Al (100 nm)	White/fluorescent	–	LT_50_ = 685/9500	^[^ ^137^ ^]^
ITO/HATCN (5 nm)/NPB (30 nm)/TCTA (10 nm)/DIC‐TRZ:10% Ir(ppy)_3_ (30 nm)/BPBiPA (40 nm)/LiF (0.5 nm)/Al (150 nm)	Green/phosphorescent	25.5	LT_90_ = 400/5000	^[^ ^299^ ^]^
ITO/HATCN (10 nm)/TAPC) (40 nm)/mCBP):emitter (15%, 15 nm)/BmPyPB (55 nm)/LiF (0.5 nm)/Al (100 nm)	Blue/phosphorescent	20.1	LT_50_ = 300/1000	^[^ ^300^ ^]^
ITO/HATCN (5 nm)/BPAPF (20 nm)/97% BPAPF:3% Ir(MDQ)2acac (15 nm)/42.5% BPAPF:42.5% SBFK:15% Ir(ppy)_3_ (10 nm)/50% BPAPF:50% SBFK (5 nm)/95% *α*,*β*‐ADN:5% DACrs (30 nm)/BPBiPA (15 nm)/LiF/Al	Blue/hybrid	31.3	LT_50_ = >7000/1000	^[^ ^301^ ^]^
ITO/2‐TNATA (60 nm)/NPB (20 nm)/*α*,*β*ADN:4% dopant (35 nm)/Alq_3_ (15 nm)/LiF (1 nm)/Al (200 nm)	Blue/fluorescent	9.25	LT_50_ = 30000/1000	^[^ ^223^ ^]^
ITO(100 nm)/triphenylamine‐containing polymer:PPBI (20 nm)/NPD (10 nm)/HTL (10 nm)/mCBP:15 wt% 4CzIPN (30 nm)/DBT‐TRZ (10 nm)/DPB:20 wt% Liq (40 nm)/Libpp (1 nm)/Al	Green/TADF	21.5	LT_50_ = 10000/1000	^[^ ^64^ ^]^
ITO/HATCN (10 nm)/NPD (40 nm)/EBL/10% PtN01N‐tBu:mCBP (25 nm)/HBL/BPyTP (40 nm)/LiF(1 nm)/Al (100 nm)	Sky blue/phosphorescent	15.9	LT_70_ = 635/1000	^[^ ^302^ ^]^
ITO (100 nm)/ HATCN (10 nm)/ Tris‐PCz (30 nm)/ mCBP (5 nm)/15 wt% of 3Ph2CzCzBN (device A) or 15 wt% of 4CzBN (device B):mCBP (30 nm)/ T2T (10 nm)/BPy‐TP2 (40 nm)/LiF (0.8 nm)/ Al (100 nm)	Sky blue/TADF	16.6	LT_90_ = 38/1000	^[^ ^303^ ^]^
ITO/HATCN (10 nm)/NPD (40 nm)/TAPC (10 nm)/*x*% Pd_3_O_3_:26mCPy (25 nm)/DPPS (10 nm)/BmPyPB (40 nm)/LiF/Al	White/phosphorescent	24.2	LT_50_ = 3000/1000	^[^ ^304^ ^]^
ITO/HATCN(10 nm)/NPD(40 nm)/Tris‐PCz(10 nm)/EML/BAlq(10 nm)/BPyTP(40 nm)/ LiF(1 nm)/Al(100 nm)	Red/phosphorescent	21.5	LT_97_ = 600/1000	^[^ ^305^ ^]^

## 5 Conclusion

OLED will become the disruptive technology in the field of display and lighting in the near future. Primarily, we gave a clear idea about the revenue of current lighting market. We discussed about the number of publications and patents in OLED field per year. From the statistics, it is very clear that many research works are going on to improve the device efficiency compared to device lifetime. It's essential to give more attention to develop long lifetime OLEDs. We covered all the extrinsic and intrinsic factor affecting the device lifetime and pointing out, how we can prevent the same. The selection of materials and designing of device architecture are strong pillars for obtaining higher lifetime and efficiency.

## Conflict of Interest

The authors declare no conflict of interest.
